# Single‐Cell Insights Into Pathogenic Profile of Orchitis and SREBP2‐Associated Cholesterol Dysregulation in Spermatogenic Failure

**DOI:** 10.1002/advs.78572

**Published:** 2026-10-31

**Authors:** Yanlin Tang, Qiaoling Jiang, Yuwen Liu, Wenxiu Zhang, Lina Cui, Xiaojian Lu, Ziyan Zhuang, Jianxing Cheng, Songzhan Gao, Yangyi Fang, Kai Hong, Jie Qiao, Xiaoyan Wang, Hui Jiang, Zhe Zhang, Jingtao Guo, Qiang Liu

**Affiliations:** ^1^ Department of Urology Center For Reproductive Medicine State Key Laboratory of Female Fertility Promotion Peking University Third Hospital Beijing China; ^2^ State Key Laboratory of Organ Regeneration and Reconstruction Institute of Zoology Chinese Academy of Sciences Beijing China; ^3^ Beijing Institute for Stem Cell and Regenerative Medicine Beijing China; ^4^ University of the Chinese Academy of Sciences Beijing China; ^5^ Department of Andrology Third Affiliated Hospital of Zhengzhou University Zhengzhou Henan China; ^6^ State Key Laboratory of Female Fertility Promotion Center For Reproductive Medicine Department of Obstetrics and Gynecology Peking University Third Hospital Beijing China; ^7^ Department of Urology Peking University First Hospital Beijing China; ^8^ The Institution of Urology Peking University Beijing China; ^9^ Beijing Key Laboratory of Urogenital Diseases (Male) Molecular Diagnosis and Treatment Center Beijing China

**Keywords:** cholesterol metabolism, macrophage, orchitis, single‐cell RNA‐seq, SREBP2

## Abstract

Orchitis, a testicular inflammation often triggered by infection, is a major cause of male infertility due to disruption of the testicular immune microenvironment and progressive loss of germ cells. Currently, no effective or targeted therapies are available. Here, we profile 66,147 single‐cell transcriptomes from testes of orchitis patients and healthy controls, complemented by histological validation. We observe profound remodeling of testicular cell composition, with marked infiltration of CD8^+^ effector T cells and inflammatory macrophages, accompanied by germ cell loss. Undifferentiated spermatogonia exhibit cell‐cycle arrest, while somatic cell populations display fibrosis, apoptosis, and loss of homeostatic functions. Notably, interstitial cells show SREBP2‐ associated reprogramming of cholesterol metabolism, characterized by increased synthesis. Inhibition of SREBP2 in a mouse orchitis model improves spermatogenesis, reduces lipid accumulation, and alleviates fibrosis. These findings demonstrate that immune activation–induced SREBP2 upregulation drives cholesterol metabolic imbalance and spermatogenic failure in orchitis, offering potential therapeutic options for inflammation‐associated male infertility.

## Graphical abstract

A first single‐cell atlas of human orchitis reveals coordinated germ‐cell loss, somatic niche dysfunction, and immune‐metabolic remodeling. Aberrant SREBP2 activation in testicular somatic cells emerges as a central pathogenic node, while Fatostatin‐mediated inhibition of the SREBP pathway improves spermatogenesis, fibrosis, and lipid accumulation in a mouse orchitis model.

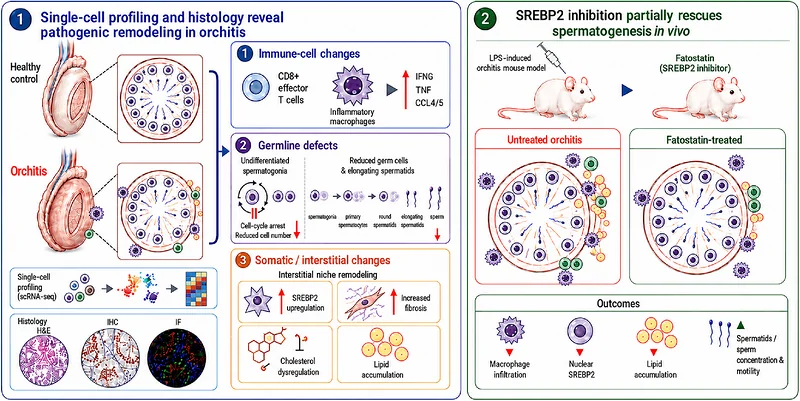

## 1 Introduction

Orchitis is a common inflammatory condition of the testes and an important contributor to male infertility. It is often caused by bacterial or viral infection and typically presents with pain, swelling, and progressive testicular atrophy [1]. Despite its clinical importance, treatment remains confined to supportive care or the use of nonspecific antibiotics and antivirals [2], which are largely ineffective in restoring fertility. The absence of targeted therapeutic strategies underscores the need to clarify the mechanisms that drive orchitis in order to develop effective interventions that safeguard male reproductive health.

A defining feature of orchitis is disruption of the testicular immune environment [1]. Under normal conditions, the testes are immune‐privileged, providing a protected niche for spermatogenesis [3]. However, this delicate immune homeostasis can be severely disrupted during infection. Evidence shows that orchitis induces increased infiltration of T cells and macrophages into the testes, accompanied by impaired spermatogenesis [4, 5]. In particular, S100A4^+^ macrophages activate complement pathways and disrupt the blood‐testis barrier (BTB), directly injuring germ cells, especially spermatogonia [6, 7, 8]. In mumps virus‐infected mice, secretion of pro‐inflammatory mediators such as TNFα and CXCL10 is markedly increased. These immune alterations can trigger severe testicular inflammation through immune cell activation and the release of inflammatory factors, ultimately leading to spermatogenic dysfunction.

Inflammation also reshapes cellular metabolism, particularly lipid and cholesterol pathways. Aberrant inflammation‐associated lipid metabolism contributes to disease progression in non‐alcoholic fatty liver disease [9], and similar processes occur in the testes. Obesity and metabolic syndrome, both characterized by chronic inflammation and oxidative stress, are major contributors to male infertility [10]. Circulating inflammatory mediators and reactive oxygen species can penetrate the testis, disrupting the finely balanced endocrine and paracrine signaling that regulates Leydig and Sertoli cell function. Such disruption impairs testosterone synthesis, compromises spermatogenesis, and damages the BTB, ultimately undermining fertility [11, 12, 13]. Multi‐omics studies of testes from patients with non‐obstructive azoospermia further highlight this correlation, showing abnormal deposition of unsaturated fatty acids, defective fatty acid metabolism in Sertoli cells, and reduced docosahexaenoic acid enrichment, all of which damage the testicular microenvironment and germ cell development [14]. These findings underscore a close, bidirectional relationship between testicular inflammation and cholesterol metabolism, yet the mechanisms linking them in orchitis remain unclear.

To address this gap, we performed single‐cell transcriptomic profiling of testis from orchitis patients and healthy controls, generating a comprehensive cellular atlas of human orchitis. Our analysis reveals extensive immune infiltration, including expansion of CD8^+^ effector T cells, alongside disrupted spermatogenesis marked by depletion of undifferentiated spermatogonia. Somatic cells also exhibited dysfunction, with myoid cells showing pronounced fibrosis and apoptosis. Importantly, we identified a shared dysregulation of cholesterol metabolism in interstitial cells, characterized by elevated expression of SREBP2, a master transcriptional regulator. Targeted inhibition of SREBP2 using Fatostatin in mouse orchitis effectively improved spermatogenesis, diminished cholesterol accumulation, and alleviated fibrosis. Taken together, these findings identify SREBP2 as a central mediator of orchitis pathogenesis and highlight it as a promising therapeutic target—not only for orchitis but potentially for a wider spectrum of inflammation‐related male infertility.

## 2 Results

### 2.1 Integrated Single‐Cell And Histological Analyses Reveal Disrupted Testicular Cell Composition in Orchitis

To build a comprehensive single‐cell atlas of orchitis testes, we performed single‐cell RNA sequencing on testicular samples collected from 4 orchitis and 4 fertile adult men (Con) (Figure 1a). Following stringent quality control, a total of 66,147 high‐quality cells were retained for downstream analysis (Figure S1a). Using Seurat, the dataset was normalized and scaled, and 2,000 highly variable genes were selected for principal component analysis (PCA) and Uniform Manifold Approximation and Projection (UMAP). The UMAP plot colored by sample identity showed good mixing of cells across samples, indicating satisfactory data integration without obvious sample‐specific batch effects (Figure S1b, d). Unsupervised clustering and canonical marker gene expression identified six major somatic cell types: endothelial cells (*VWF* ^+^), lymphocyte cells (*CD3D* ^+^, *CD79A* ^+^), myeloid cells (*C1QA* ^+^), myoid/interstitial cells (*PDGFRA* ^+^), Sertoli cells (*SOX9* ^+^), and smooth muscle cells (*NOTCH3* ^+^). In addition, three primary germ cell populations were defined: spermatogonia (*UTF1* ^+^), spermatocytes (*SYCP3* ^+^), and spermatids (*ACRV1* ^+^, *PRM3* ^+^) (Figure 1b, g). Our STARTRAC‐dist index revealed distinct patterns, characterized by a significant expansion of lymphocytes and myoid/interstitial cells in orchitis, accompanied by a marked reduction in germ cells compared to controls (Figure 1c). The number and percentage of each annotated major cell type in every individual sample are provided in Table S1. These molecular findings were strongly corroborated by histological staining (H&E), which showed extensive germ cell loss and interstitial expansion in orchitis testes (Figure 1d). Moreover, the immunofluorescence (IF) staining revealed the compositional change of immune cells, with an expansion of CD8^+^ T cells and macrophages (Figure 1e,f). Consistent with these results, orchitis patients exhibited reduced testicular volume and abnormal follicle‐stimulating hormone (FSH, normal range: 0.7‐11.1 IU/L) and luteinizing hormone (LH, normal range: 0.8‐7.6 IU/L) levels, further supporting the presence of severe testicular dysfunction (Figure S1c).

**FIGURE 1:**
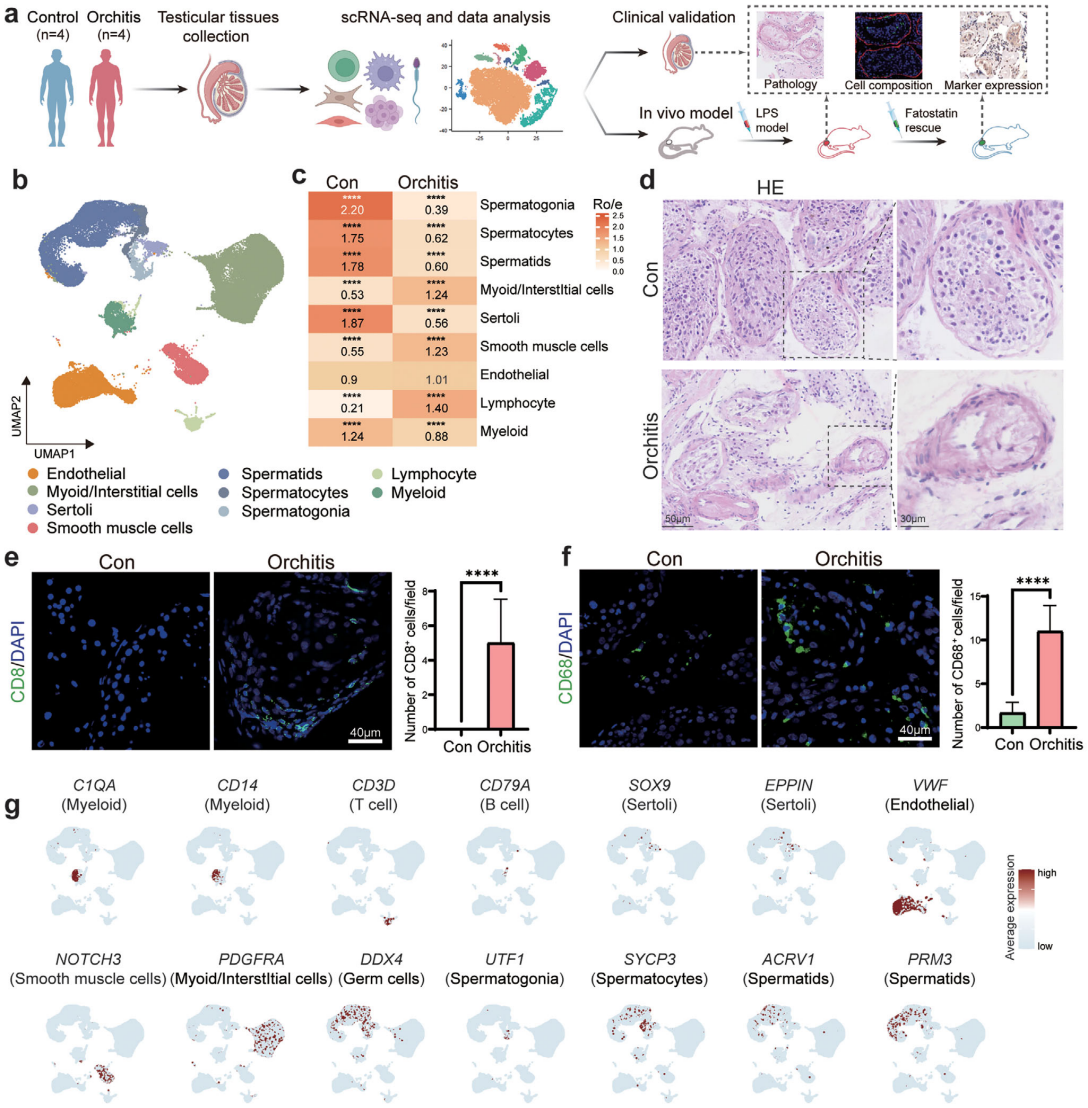
Single‐cell transcriptomic atlas of testicular cells from healthy men and orchitis. a. Schematic illustration of the research workflow. b. Uniform manifold approximation and projection (UMAP) plot of the testis sample from controls (n = 4) and orchitis (n = 4). Dots represent individual cells, and colors represent different cell types. c. Tissue preference of each cluster was estimated using the STARTRAC‐dist index. Ro/e represents the ratio of the observed to the expected cell number. Numbers denote Ro/e values. Significance was assessed using two‐sided Fisher's exact tests with BH‐FDR correction (*FDR < 0.05, ****FDR < 0.0001). d. Hematoxylin and eosin (H&E) staining of a control testis section (top) and an orchitis testis section (bottom) with corresponding magnifications. Scale bar, 50 µm (left) and 30 µm (right). e. Left: IF staining of CD8 in tissue sections of the controls and orchitis. Right: Quantification of the number of CD8^+^ cells per field in the control and orchitis groups. Bars represent the mean with SD, n = 3. The p value was calculated by Student's t test. **** *p* < 0.0001. Scale bar, 40 µm. f. Left: IF staining of CD68 in tissue sections of the controls and orchitis. Right: Quantification of the number of CD68^+^ cells per field in the control and orchitis groups. Bars represent the mean with SD, n = 3. The p value was calculated by Student's t test. **** *p* < 0.0001. Scale bar, 40 µm. g. UMAP plot presenting the expression pattern of selected markers identifying each cell type. Color gradients (red) represent average expression level as shown in the color legend to the right.

### 2.2 Pro‐Inflammatory Immune Cells Infiltrate the Testes in Orchitis

To define the immune alterations underlying the inflammatory nature of orchitis, we reclustered the lymphocyte population. Based on marker genes, we identified six subclusters, including CD8^+^ T effector cells (CD8^+^ Teff, *CD8A* ^+^, *GZMA* ^+^), proliferating CD8^+^ T cells (prolif CD8^+^ T, *CD8A* ^+^, *MKI67* ^+^), Treg (*FOXP3* ^+^, *IL2RA* ^+^), natural killer cells (NK, *NCAM1* ^+^, *NKG7* ^+^), B cells (*CD79A* ^+^, *IGHM* ^+^), and T helper cells (Th, *CD40LG* ^+^, *ICOS* ^+^) (Figure 2a,c). In orchitis samples, CD8^+^ Teff cells dominated the lymphocyte compartment, in contrast to their near absence in controls, while B cells and Tregs were also enriched in orchitis (Figure 2b). Consistent with their central role in inflammation, CD8^+^ Teff cells displayed significantly higher activation scores (Figure S2a) and, together with NK cells, showed elevated cytokine production and cytolytic activity (Figure 2d). At the transcriptomic level, CD8^+^ Teff cells upregulated key pro‐inflammatory mediators—including *CCL4*, *CCL5*, *IFNG*, and *TNF* (Figure 2e)—and Gene Ontology (GO) analysis confirmed enrichment for pathways related to T cell activation and cytokine secretion in orchitis (Figure S2b). These findings suggest CD8^+^ Teff cells as major drivers of inflammatory cascades within the orchitis testicular microenvironment.

**FIGURE 2:**
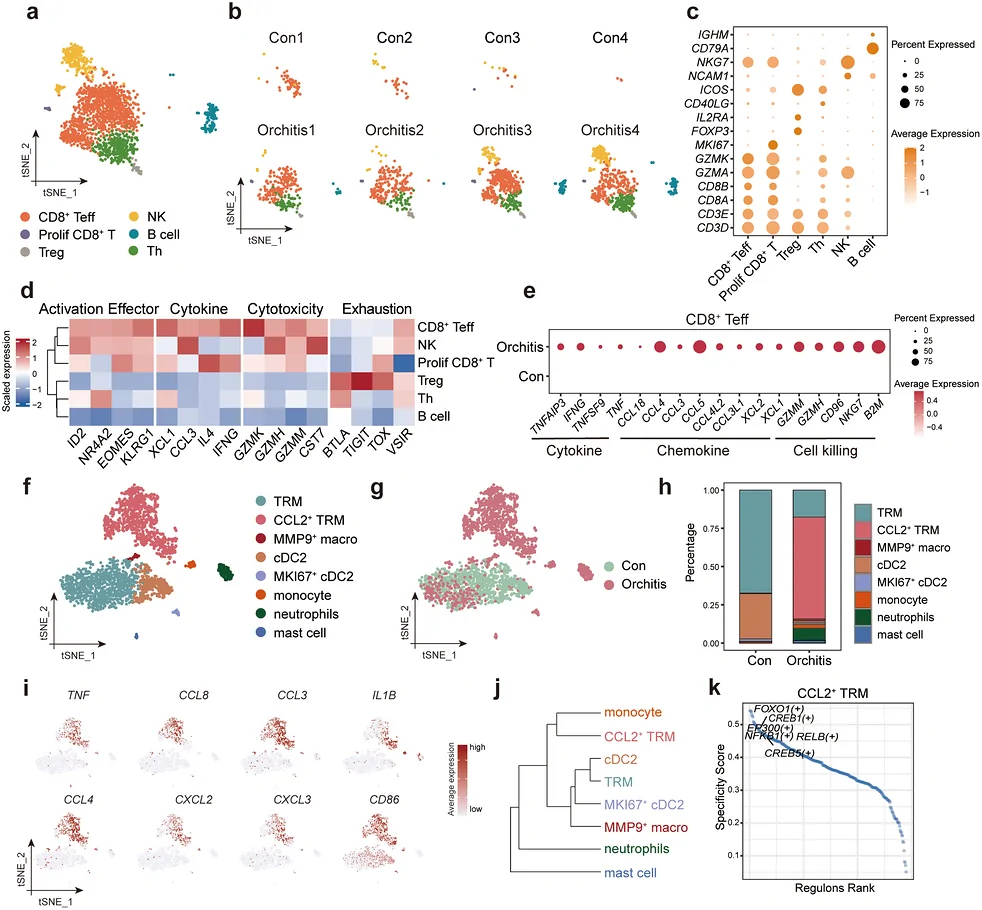
Pro‐inflammatory immune cells infiltrate the testes of orchitis. a. T‐distributed stochastic neighbor embedding (t‐SNE) plot of lymphocytes from all samples. Different colors indicate different subtypes. b. Separate t‐SNE plots of lymphocytes in each sample. c. Dot plot presenting the expression level of selected markers in each lymphocyte. Dot size represents the expression ratio of each gene and the gradient of orange color represents the average expression level of the genes. d. Expression pattern of genes related to different immune functions in each lymphocyte subtype. e. Dot plot presenting the differential expression of markers related to cytokine, chemokine and cell killing in CD8^+^ Teff cells between the control and orchitis groups. f. t‐SNE plot of myeloid cells from all samples. Different colors indicate different subtypes. g. The distribution of myeloid cells in the control and orchitis group in the form of t‐SNE plot. h. The proportion of myeloid cells in the control and orchitis group. i. The expression pattern of the markers of CCL2^+^ TRM cells in the form of t‐SNE plot. Color gradients (red) represent average expression level as shown in the color legend to the right. j. Dendrogram representing the transcriptional clustering of the myeloid cells. k. The regulons rank in CCL2^+^ TRM cells according to specificity score.

Myeloid cells, the most abundant immune population in the testis [15], also showed marked remodeling in orchitis. Reclustering revealed eight subpopulations (Figure 2f, Figure S2c). In healthy controls, tissue‐resident macrophages (TRM) and type II classical dendritic cells (cDC2) predominated, whereas in orchitis, TRM cells were largely replaced by inflammatory CCL2^+^ TRM cells unique to disease samples (Figure 2g‐h). These CCL2^+^ TRM cells were characterized by a strong pro‐inflammatory and chemotactic profile. They showed specific expression of pro‐inflammatory cytokines such as *TNF* and *IL1B*, along with multiple chemokines including *CCL8*, *CCL4*, and *CXCL3* (Figure 2i). The selective expression of the M1 macrophage marker *CD86* further confirmed their inflammatory nature. GO analysis showed that genes highly expressed in CCL2^+^ TRM cells were significantly enriched in pathways related to monocyte and lymphocyte recruitment (Figure S2e), highlighting their role in attracting other immune cells to the site of inflammation. PCA analysis confirmed that CCL2^+^ TRM cells were transcriptionally distinct from TRM cells (Figure S2d). Further analysis showed that these cells have a transcriptional signature more similar to monocytes than to healthy TRM cells (Figure 2j), suggesting they acquire monocyte‐like features during inflammation. Consistently, inflammatory response scoring demonstrated that CCL2^+^ TRM cells, together with monocytes and neutrophils, exhibited the highest inflammatory activity, exceeding that of TRM cells (Figure S2f). Furthermore, SCENIC analysis identified key pro‐inflammatory transcription factors—NFKB1, CREB1, and EGR1—as central regulators of CCL2^+^ TRM cells (Figure 2k, Figure S2g). These TFs are well‐established enhancers of *CCL2* expression [16, 17], further linking this population to chemokine‐driven immune recruitment. These findings suggest that the emergence of CCL2^+^ TRM cells represents a functionally specialized inflammatory macrophage subset in orchitis, orchestrating chemokine‐mediated recruitment of other immune cells and amplifying local inflammation.

In contrast, TRM cells from healthy controls were enriched for complement activation and homeostatic functions (Figure S2e), underscoring their role in maintaining tissue integrity under physiological conditions. Differential expression analysis further highlighted the functional divergence between controls and orchitis (Figure S2h). Control TRM cells exhibited elevated expression of pattern recognition receptors, including *TLR2* and *TLR4* (Figure S2j), indicative of enhanced innate immune surveillance [18, 19]. Consistently, GO enrichment analysis revealed upregulation of pathways related to phagocytosis and NOD‐like receptor signaling (Figure S2i), functions central to tissue maintenance and immune homeostasis [20]. In orchitis, these protective programs were markedly attenuated, coinciding with a pronounced influx of inflammatory immune populations. Collectively, these findings suggest that the loss of homeostatic TRM functions, together with the emergence of inflammatory CCL2^+^ TRM cells, may underlie immune imbalance and contribute to the pathogenesis of testicular inflammation in orchitis.

Given the marked infiltration of CD8^+^ Teff cells and CCL2^+^ TRM cells, along with elevated chemokine expression in orchitis, we next investigated how cell–cell communication might shape the testicular microenvironment. Both the number and strength of interactions were markedly elevated in orchitis compared with controls (Figure S3a–b), consistent with a hyperactive immune state. Further examination of intercellular signaling pathways showed that multiple classical immune signaling axes—such as IL‐1, LCK, and IFN‐II—were uniquely activated in orchitis but absent in controls (Figure S3c). Notably, CXCL signaling was found to be selectively upregulated in orchitis (Figure S3d), promoting immune cell recruitment via chemokine receptor engagement and activation of intracellular migration programs that direct immune cells toward inflamed tissue [20]. A key ligand–receptor axis was identified in orchitis: CCL2^+^ TRM cells and monocytes secreted CXCL ligands predicted to recruit neutrophils, which specifically expressed *CXCR1* and *CXCR2* (Figure S3e). As innate immune “first responders”, neutrophils rapidly migrate to inflammatory sites and exert antimicrobial functions through degranulation [21, 22]. In line with this, neutrophils were markedly increased in orchitis testes, which likely exacerbates tissue damage, significantly contributing to the overall pathology and severity of orchitis. The dysregulated recruitment of neutrophils, orchestrated by the CXCL‐CXCR axis, thus plays a central role in driving the inflammatory and damaging processes observed.

### 2.3 Molecular Basis of the Impaired Spermatogenesis in Orchitis

To assess the impact of orchitis on germ cell development, we compared the molecular features and abundance of germ cell subpopulations between orchitis and control testes. Reclustering of germ cells identified six subtypes representing a continuous developmental trajectory from undifferentiated spermatogonia to elongated spermatids (Figure 3a, b). While this trajectory was preserved, a prior analysis of the overall cell proportions in orchitis testes had already shown an overall reduction in germ cell numbers (Figure 1c). Further investigation revealed that this decline was most pronounced among the undifferentiated spermatogonia and the elongating spermatids (Figure 3c). This specific pattern of loss suggests a profound disruption in spermatogenesis, affecting both the stem cell population at the beginning of the process and the mature cell population at its conclusion.

**FIGURE 3:**
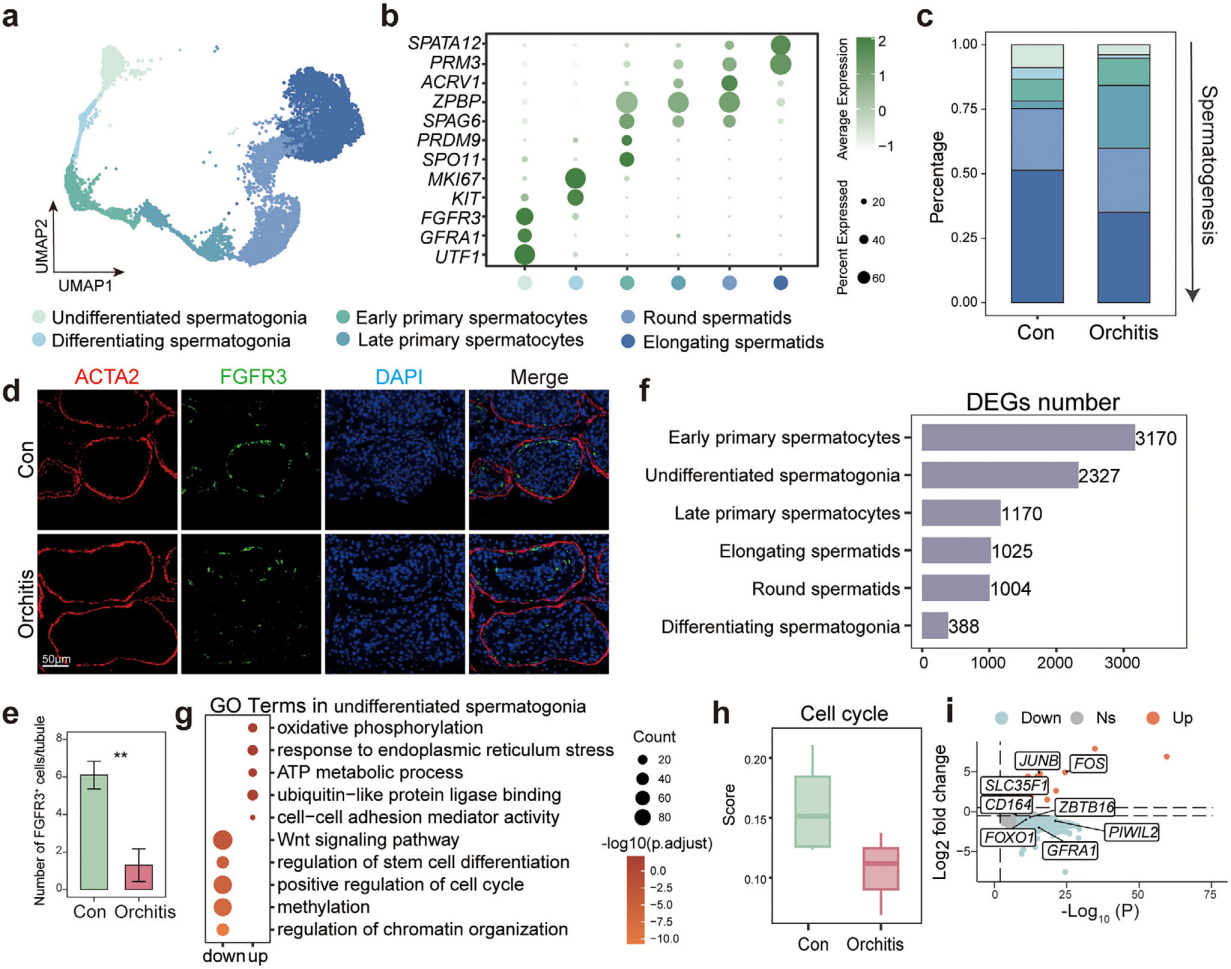
Impaired spermatogenesis in orchitis. a. UMAP plot displaying the spermatogenic cells in all samples. Different colors represent different cell types. b. Dot plots presenting the expression of markers related to different germ cells. Color gradients (green) represent the expression levels and dot size represents the expressed percentage. c. Bar plot showing the percentage of germ cells, and colors represent different germ cell subtypes. d. IF staining of FGFR3 and ACTA2 in tissue sections of the controls and orchitis, showing the number of FGFR3^+^ cells significantly decreased in the orchitis group. Scale bar, 50 µm. e. Quantification of the number of FGFR3^+^ cells per spermatogenic tubule in the control and orchitis groups. Bars represent the mean with SD of 30 independent tubules in the control (n = 3) and orchitis groups (n = 3). The *p* value was calculated by Student's t test. **p < 0.01. f. Bar plot showing the number of DEGs in different germ cells between controls and orchitis. g. Downregulated (left) and upregulated (right) GO terms enriched in undifferentiated spermatogonia cells of the orchitis group compared to the controls. h. Bar plot showing the signature score of cell cycle between the control and orchitis groups. i. Volcano plot presenting the differentially expressed genes of undifferentiated spermatogonia cells between the controls and orchitis group. Each dot represents one gene and different colors represent different expression patterns as shown in the legend at the top.

As the initiating population in spermatogenesis, undifferentiated spermatogonia provide the self‐renewal and differentiation capacity required to sustain germ cell production [23]. Consistent with their depletion, IF staining revealed a striking reduction of FGFR3^+^ undifferentiated spermatogonia in orchitis compared with controls (Figure 3d,e). To more precisely quantify the cellular changes in orchitis, we analyzed the number of differentially expressed genes (DEGs) for each germ cell subtype and found that undifferentiated spermatogonia showed a significantly greater number of DEGs than most other subtypes (Figure 3f). We next identified genes differentially expressed between orchitis and control groups and performed GO enrichment analysis for undifferentiated spermatogonia. In controls, undifferentiated spermatogonia were significantly enriched for pathways governing stem cell differentiation and cell cycle—processes essential for maintaining spermatogonial stem cell function—whereas in orchitis testes, they showed enrichment for pathways associated with oxidative phosphorylation and endoplasmic reticulum stress responses (Figure 3g). Consistently, the cell cycle scores exhibited a significant reduction in orchitis compared to the controls (Figure 3h). Importantly, *CD164*, a gene previously linked to spermatogonial apoptosis in non‐obstructive azoospermia [24], was also significantly upregulated in orchitis. Together, these findings demonstrate that orchitis profoundly disrupts the maintenance and functional integrity of undifferentiated spermatogonia, thereby undermining the foundation of spermatogenesis and driving widespread germ cell loss.

### 2.4 Disrupted Spermatogenic Niche and Interstitial Cell Dysfunction in Orchitis

To investigate alterations in somatic cell populations in orchitis, we isolated Sertoli cells, myoid/interstitial cells, and re‐clustered them, identifying mesenchymal cells (*H19* ^+^, *PDGFRA* ^+^, *IGF1* ^+^, *DLK1* ^+^), myoid cells (*ACTA2* ^+^, *WFDC1* ^+^), Sertoli cells (*SOX9* ^+^), and Leydig cells (*CYP17A1* ^+^) (Figure 4a, c). Cell counts showed marked reductions of both Sertoli and myoid cells in orchitis (Figure 4b), a finding further validated by IF staining (Figure 4d–e, Figure S4a,b). Sertoli cells, the only somatic cell type located within seminiferous tubules, are essential for coordinating and maintaining testicular homeostasis through androgen receptor (AR) signaling and phagocytosis [25, 26]. In orchitis, AR signature scores showed a modest decline compared with controls (Figure S4c), while genes related to phagocytosis—another critical Sertoli cell function—displayed a disrupted expression pattern (Figure S4d).

**FIGURE 4:**
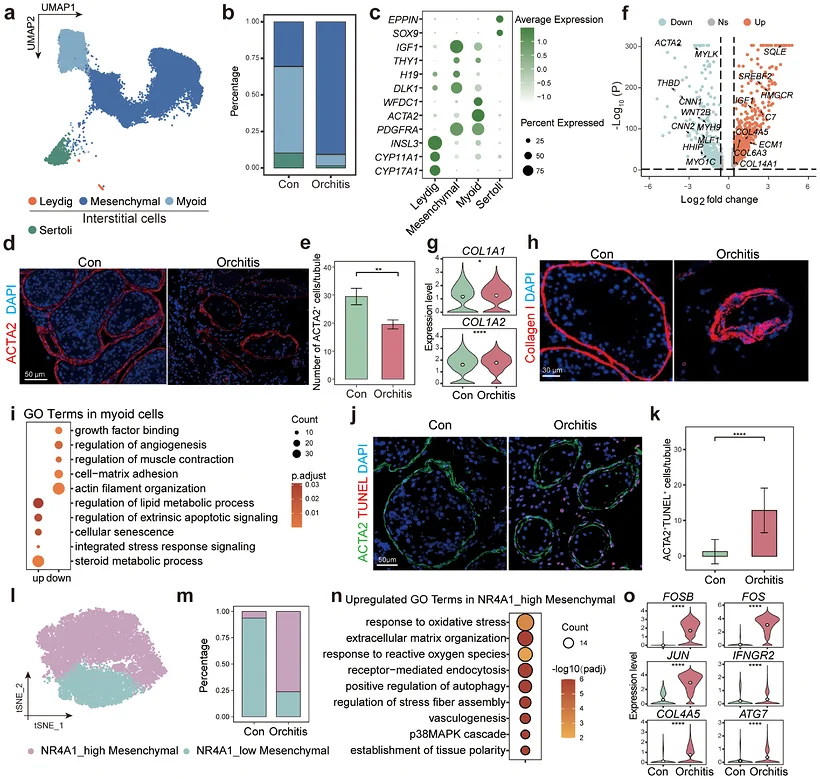
Disrupted spermatogenic niche and interstitial cell dysfunction in orchitis. a. UMAP plot displaying the somatic cells in all samples. Different colors represent different cell types. b. Bar plot showing the percentage of somatic cells in controls and orchitis. c. Dot plots presenting the expression of markers related to different somatic cells. Color gradients (green) represent the expression levels and dot size represents the expressed percentage. d. IF staining of ACTA2 (red) in the tissue section of the control and orchitis group. Scale bar, 50 µm. e. Quantification of the number of ACTA2^+^ cells per spermatogenic tubule in the control and orchitis groups. Bars represent the mean with SD of 30 independent tubules in the control (n = 3) and orchitis groups (n = 3). The *p* value was calculated by Student's t test. **p < 0.01. f. Volcano plot presenting the differentially expressed genes of myoid cells between the control and orchitis group. g. Violin plot showing the differential expression of *COL1A1* (top) and *COL1A2* (bottom) between the control and orchitis groups. The *p* value was calculated by Wilcoxon test. ****p < 0.0001.h. IF staining of Collagen I (red) in the tissue section of the control and orchitis group. Scale bar, 30 µm. i. Dot plot displaying the GO terms enriched in the myoid cells of the orchitis group compared to the control group. The color gradients represent the *p* value and the dot sizes represent the enriched gene counts. j. IF staining of ACTA2 (green) and TUNEL (red) in the tissue section of the control and orchitis group. Scale bar, 50 µm. k. Quantification of the number of ACTA2^+^TUNEL^+^ cells per spermatogenic tubule in the control and orchitis groups. Bars represent the mean with SD of 30 independent tubules in the control (n = 3) and orchitis groups (n = 3). The p value was calculated by Student's t test. ****p < 0.0001. l. t‐SNE plot displaying the NR4A1_high mesenchymal cells and NR4A1_low mesenchymal cells in all samples. Different colors represent different cell types. m. Bar plot showing the percentage of NR4A1_high mesenchymal cells and NR4A1_low mesenchymal cells in controls and orchitis. n. Dot plot displaying the upregulated GO terms enriched in NR4A1_high mesenchymal cells. o. Violin plots showing the expression of representative upregulated genes in NR4A1_high mesenchymal cells from control and orchitis groups. The *p* value was calculated using the Wilcoxon test. **** *p* < 0.0001.

Myoid cells are responsible for transporting sperm and tubular fluid, as well as supporting the spermatogonial stem cell niche through secretion of growth factors and extracellular matrix (ECM) proteins [27]. To assess their alterations in orchitis, we compared transcriptional profiles of myoid cells from orchitis and controls. Fibrosis‐associated genes, including *COL14A1* and *COL6A3*, together with the ECM protein *ECM1*, were significantly upregulated in orchitis (Figure 4f). IF staining further confirmed an accumulation of ECM1 and Collagen I in orchitis testes (Figure 4 h, Figure S4e,f), indicating fibrotic remodeling and ECM dysregulation. Fibrosis is a common feature of male reproductive disorders and is often triggered by cellular stress, which can compromise reproductive function [28, 29, 30]. GO enrichment analysis of upregulated genes in orchitis myoid cells revealed associations with apoptosis and lipid metabolism (Figure 4i), suggesting stress‐induced vulnerability and loss of these cells. Consistent with this, TUNEL assays confirmed significantly increased apoptosis in orchitis myoid cells (Figure 4j–k, Figure S4j). Together, these findings demonstrate that fibrosis, ECM abnormalities, and stress responses converge to drive myoid cell dysfunction, potentially undermining seminiferous tubule integrity and disrupting testicular homeostasis.

We observed a substantial increase of mesenchymal cells in orchitis. To further dissect their alterations, we performed unsupervised reclustering of the annotated mesenchymal cells and identified two transcriptionally distinct subclusters (Figure 4l). Subsequent examination of their gene expression profiles showed higher NR4A1 and EGR1 expression in one mesenchymal subcluster (Figure S4g), two immediate‐early genes previously implicated in stress responses and fibrosis [31, 32, 33]. We therefore provisionally refer to the subclusters as NR4A1_high and NR4A1_low mesenchymal cells, while these names describe their expression profiles rather than markers used to define mesenchymal cell identity. We observed a notable increase in the proportion of NR4A1_high mesenchymal cells in orchitis compared to controls (Figure 4m). By performing GO analysis, enrichments including oxidative stress and ECM assembly pathways were significantly upregulated in orchitis NR4A1_high mesenchymal cells (Figure 4n). Furthermore, this specific cell population showed a significant increase in the number of DEGs in orchitis (Figure S4h). This was accompanied by a higher expression of the fibrosis‐related gene *COL4A5* (Figure 4o), while genes involved in complement activation, such as *C3* and C7 [34 *]*, were downregulated (Figure S4i). Together, these findings suggest that NR4A1_high mesenchymal cells represent an activated, stress‐responsive fibroinflammatory state that may actively contribute to ECM remodeling and fibrosis in orchitis.

### 2.5 SREBP2‐Associated Cholesterol Accumulation in Interstitial Cells of Human and Mouse Orchitis

We found that 34.4% of DEGs were shared between myoid cells and mesenchymal cells (Figure 5a), suggesting a convergent transcriptional response to orchitis. Upregulated GO terms in both cell types (hereafter referred to as interstitial cells) were enriched for cholesterol metabolism and steroid biosynthesis pathways (Figure 5b), indicating a global metabolic dysregulation. Key cholesterol metabolism genes, including *SREBF2*, *LDLR*, and *HMGCR*, were markedly upregulated in interstitial cells from orchitis, with consistently higher expression across individual orchitis samples than in controls (Figure 5c, Figure S5a,b). At transcriptional level, *SREBF2*, a master transcriptional regulator of cholesterol metabolism, controls *LDLR*‐mediated cholesterol uptake and *HMGCR*‐driven biosynthesis [35, 36], and its dysregulation has been associated with immune activation and disrupted cholesterol homeostasis [37, 38]. These findings suggest that elevated *SREBF2* may drive the observed cholesterol metabolic reprogramming in orchitis. To further validate these findings, we performed gene set scoring for cholesterol synthesis and metabolism pathways, interstitial cells from orchitis exhibited significantly higher scores than controls, indicating an actively upregulated state of cholesterol metabolism (Figure 5d, Figure S5c). We next validated these findings using immunohistochemistry (IHC). The data confirmed significantly elevated expression of SREBP2 (encoded by *SREBF2*) and LDLR in orchitis compared to controls (Figure 5e–g, Figure S5d). In contrast, the expression of ABCA1, a key efflux transporter facilitating cholesterol export [39], was notably reduced in the orchitis (Figure S5e,f). Notably, loss of ABCA1 has been shown to cause lipid accumulation within the testis, accompanied by decreased intratesticular testosterone levels and reduced sperm counts [39]. These results together suggested that interstitial cells in orchitis may undergo SREBP2‐ associated cholesterol metabolic reprogramming, characterized by increased synthesis and uptake and reduced efflux.

**FIGURE 5:**
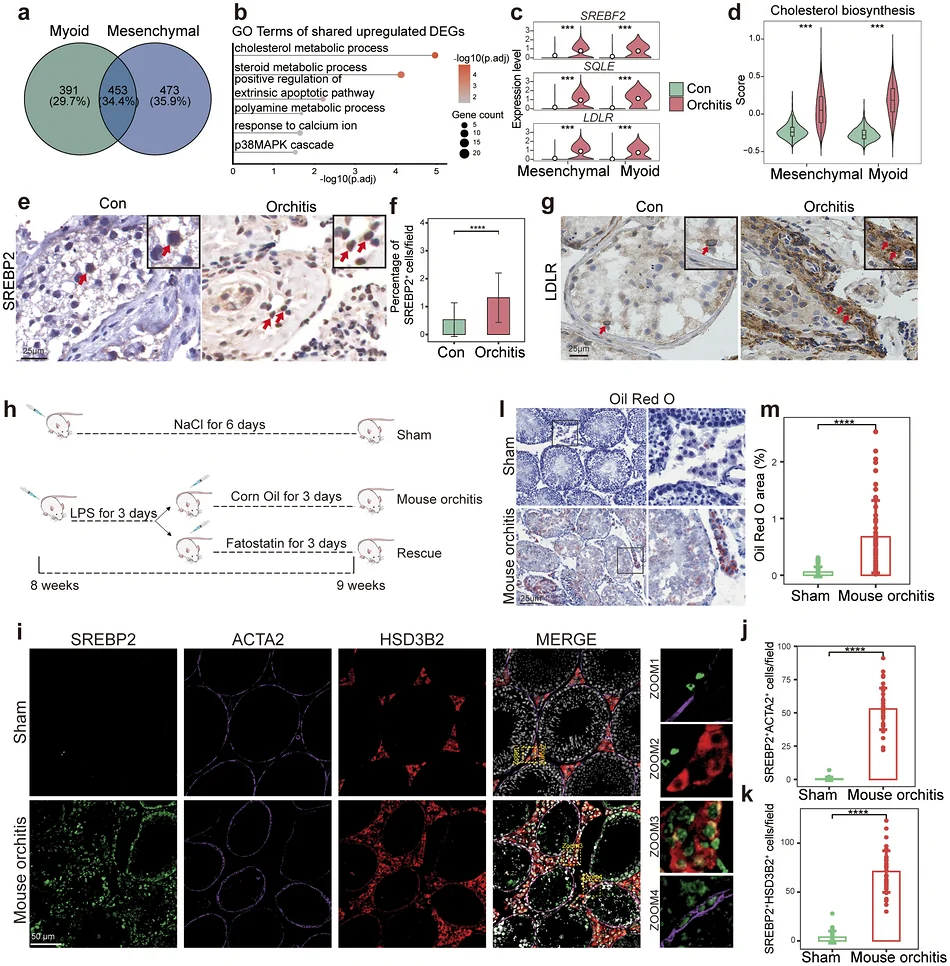
SREBP2‐ associated cholesterol accumulation in the interstitial cells of human and mouse orchitis. a. Venn plot presenting the numbers of DEGs of the myoid cells and mesenchymal cells. b. Lollipop plot showing the GO terms enriched by the shared upregulated DEGs of the myoid cell and mesenchymal cells in the orchitis group. c. Violin plot displaying the differential expressions of *SREBF2*, *SQLE*, and *LDLR* in mesenchymal and myoid cells between the control and orchitis groups. The *p* value was calculated by Wilcoxon test. ***p < 0.001. d. Violin plot presenting the calculated scores of cholesterol biosynthesis in mesenchymal and myoid cells between the control and orchitis groups. The *p* value was calculated by Student's t test. ***p < 0.001. e. Immunohistochemical (IHC) staining of SREBP2 in the tissue section of the control and orchitis group. Enlarged images and arrowheads indicate the SREBP2 positive cells. Scale bar, 25 µm. f. Quantification of the number of SREBP2^+^ cells per field in the control and orchitis groups. Bars represent the mean with SD of 50 independent fields in the control (n = 8) and orchitis groups (n = 8). The *p* value was calculated by Student's t test. **p < 0.01. g. The expression of LDLR in the interstitial cells of both the control and orchitis groups shown by IHC staining. Enlarged images and arrowheads indicate the LDLR positive cells. Scale bar, 25 µm. h. The flow chart of animal experiments. i. IF staining of SREBP2 (green), ACTA2 (purple) and HSD3B2 (red) in the tissue section of sham and mouse orchitis groups. Scale bar, 50 µm. j‐k. Quantification of the SREBP2+ myoid cells and SREBP2+ mesenchymal cells per field in the sham (n = 13) and mouse orchitis groups (n = 8). The *p* value was calculated by Student's t test. ***p < 0.001. l. Oil red O staining of lipid droplets in the tissue sections of the sham and mouse orchitis groups. Scale bar, 25 µm. m. Quantification of the oil red O area in the sham (n = 13) and mouse orchitis group (n = 8). The *p* value was calculated by Student's t test. ****p < 0.0001.

In interstitial cells of orchitis, we observed a dysregulation of cholesterol metabolism characterized by an increase of SREBP2, which we speculated might contribute to spermatogenic dysfunction in orchitis. To test this hypothesis, we designed an in vivo experiment using Fatostatin to inhibit SREBP2 and evaluate its therapeutic effect on fertility and associated pathological phenotypes (Figure 5h). Our initial step was to confirm that the mouse orchitis model accurately replicated the pathological features of orchitis observed in our study. H&E and Masson's staining showed that mouse orchitis testes displayed severe architectural disruption of seminiferous tubules, including tubular shrinkage, massive immune infiltration, peritubular fibrosis, and extensive germ cell loss compared with the sham group (Figure S5g), closely resembling orchitis pathology (Figure 1d). Further investigation with F4/80 staining confirmed an increased infiltration of macrophages in mouse orchitis (Figure S5h‐i), a key indicator of inflammation, particularly within the seminiferous tubules, suggesting a disruption of the testicular immune barrier. Furthermore, both IF and CASA‐based sperm analyses confirmed significantly impaired fertility in mouse orchitis. Specifically, PNA staining revealed a marked reduction in acrosome‐positive spermatids, reflecting significantly decreased sperm counts (Figure S5j‐k), while CASA demonstrated profound defects in sperm concentration and motility (Figure S5l). Consistently, mouse orchitis testes exhibited significantly reduced testis weight and testis‐to‐body weight ratios compared with the sham group (Figure S5m,n), indicative of severe testicular atrophy. At the protein level, IF validation showed robust upregulation of SREBP2 in interstitial cells of mouse orchitis testes, with ACTA2 and HSD3B2 used to localize myoid cells and mesenchymal cells respectively (Figure 5i–k), accompanied by a marked increase in lipid droplet deposition (Figure 5l–m). Collectively, these findings demonstrate that the mouse orchitis model recapitulates key pathological features of orchitis, including impaired spermatogenesis, testicular atrophy, and dysregulated cholesterol metabolism associated by SREBP2 upregulation.

### 2.6 Inhibition of SREBP2 Improved Spermatogenesis in Mouse Orchitis

After establishing a mouse model that closely mimics the key pathological features of orchitis, we investigated whether inhibiting SREBP2 could rescue spermatogenesis and associated testicular damage. We treated the mouse orchitis group with Fatostatin, a known SREBP2 inhibitor [40, 41, 42], to generate a “rescue” cohort. In testes of the rescue group, H&E staining showed pronounced morphological improvement, characterized by reduced immune cell infiltration and a notable restoration of the seminiferous epithelium (Figure 6a, Figure S6e). F4/80 staining confirmed that the number of macrophages was reduced in the rescue group, which also indicated that inflammation was alleviated (Figure 6e). In addition, the number of PNA‐positive spermatids was markedly increased compared to the untreated mouse orchitis group (Figure 6b–c), indicating a partial restoration of fertility development. Consistent with this, CASA analysis revealed substantial improvements in both sperm concentration and motility in the rescue group, confirming a functional restoration of spermatogenesis (Figure 6d). In parallel, we observed a significant increase in both testis weight and the testis‐to‐body weight ratio following SREBP2 inhibition (Figure S6a,b), reflecting a partial recovery of testicular structure and overall reproductivity. Histological analysis further demonstrated the therapeutic effects of SREBP2 inhibition. Masson's trichrome staining revealed a marked reduction in interstitial fibrosis (Figure S6c), a key hallmark of chronic inflammation. Consistent with our previous findings on cholesterol metabolism, Oil Red O staining revealed smaller interstitial and larger intratubular lipid droplets in mouse orchitis testes, and both patterns were less prominent after Fatostatin treatment, although the cells containing the intratubular droplets could not be identified from this staining alone. (Figure 6f‐g). Meanwhile, the increased total cholesterol level in mouse orchitis group was also normalized by Fatostatin treatment (Figure S6d), highlighting the efficacy of SREBP2 suppression in normalizing testicular metabolic homeostasis.

**FIGURE 6:**
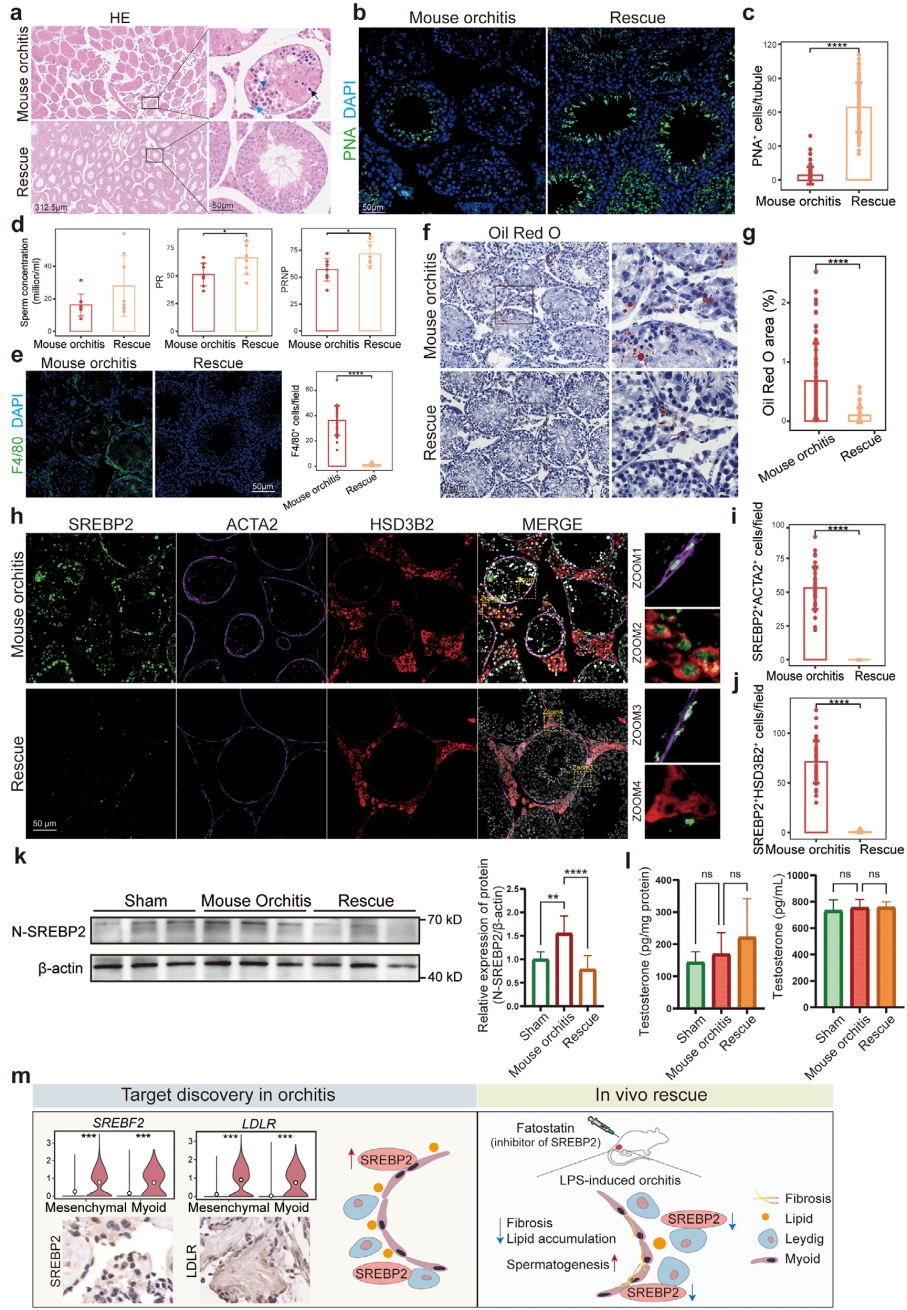
Inhibition of SREBP2 improved spermatogenesis in mouse orchitis. a. H&E staining of mouse orchitis testis (n = 8) section (top) and rescue testis (n = 8) section (bottom) with corresponding magnifications. Scale bar, 312.5 µm (left) and 50 µm (right). Black arrows point to lymphocytes; blue arrows point to myeloid cells. b. IF staining of PNA (green) in the tissue section of mouse orchitis (n = 8) and rescue groups (n = 8). le bar, 50 µm. c. Quantification of PNA^+^ cells per spermatogenic tubules in both mouse orchitis (n = 8) and rescue groups (n = 8). The *p* value was calculated by Student's t test. ****p < 0.0001. d. Histogram demonstrating the differences of sperm quality indicators (sperm concentration, PR, PRNP) between the mouse orchitis (n = 8) and rescue groups (n = 8). PR, progressive motility. PRNP, progressive and non‐progressive motility. The p value was calculated by Student's t test. *p < 0.05. e. Left: IF staining of F4/80^+^ macrophage cells (green) in the tissue section of mouse orchitis and rescue groups. Scale bar, 50 µm. Right: Quantification of the F4/80^+^ macrophage cells in the mouse orchitis (n = 8) and rescue group (n = 8) f. Oil red O staining of lipid droplets in the tissue sections of the orchitis and rescue groups with one magnification in each section. Scale bar, 25 µm. g. Quantification of the oil red O area in the mouse orchitis (n = 8) and rescue group (n = 8). h. IF staining of SREBP2 (green), ACTA2 (purple) and HSD3B2 (red) in the tissue section of mouse orchitis and rescue groups. i‐j. Quantification of the SREBP2^+^ myoid cells and SREBP2^+^ mesenchymal cells in the mouse orchitis (n = 8) and rescue groups (n = 8). The *p* value was calculated by Student's t test. ****p < 0.0001. k. Representative western blot images (Left) and the corresponding quantification (Right) of N‐SREBP2 and β‐actin in testicular tissue from the sham, mouse orchitis and rescue groups, n = 3 per group. Data are presented as mean ± SD and analyzed by One‐way ANOVA. **, *p* < 0.01; ****, *p* < 0.0001. l. Histogram displaying the intratesticular (left) and serum (right) testosterone levels in the sham, mouse orchitis and rescue groups (n = 5–6). The p value was calculated by One‐way ANOVA. ns, not significant. m. Schematic diagram representing the role of SREBP2 in orchitis.

Furthermore, IF staining detected SREBP2 in interstitial cells and some basal intratubular cells in mouse orchitis testes, though the latter could not be assigned to a specific cell type without co‐staining. Following Fatostatin treatment, SREBP2 expression in interstitial cells, especially the nuclear expression, was significantly diminished in the rescue group (Figure 6h–j). Meanwhile, Western blotting (WB) demonstrated the activated form of SREBP2, known as nuclear SREBP2 (N‐SREBP2), was significantly increased in the mouse orchitis group compared with the sham group, whereas Fatostatin treatment markedly reduced N‐SREBP2 abundance relative to the untreated orchitis group (Figure 6k), confirming the pathogenic role and effective pharmacological targeting of this transcriptional regulator. Besides, serum and intratesticular testosterone levels did not differ significantly among the sham, mouse orchitis, and rescue groups at the measured time point (Figure 6l). Thus, neither LPS exposure nor Fatostatin treatment produced a detectable change in testosterone levels under these experimental conditions, and the phenotypic improvement following Fatostatin treatment is unlikely to be explained by restoration of testosterone levels. Overall, these results demonstrate that inhibiting SREBP2 not only improves spermatogenic defects but also mitigates fibrosis and cholesterol dysregulation in the testis (Figure 6m).

## 3 Discussion

In this study, we established a comprehensive single‐cell atlas of testicular tissues from patients with orchitis‐induced NOA and delineated coordinated alterations across germcell, somatic, and immune compartments. These findings reveal that chronic post‐orchitis NOA is characterized by extensive remodeling of the testicular microenvironment, involving impaired germ‐cell maintenance, inflammatory activation, and metabolic dysregulation. Together, our data identify an immune–metabolic axis that may contribute to persistent testicular dysfunction following orchitis‐associated injury.

The loss of undifferentiated spermatogonia represents a critical disruption to the foundation of spermatogenesis, as these cells maintain the capacity for self‐renewal and continuous germ‐cell production. In parallel, fibrosis and apoptosis in peritubular myoid cells indicate profound remodeling of the somatic niche, which may compromise seminiferous tubule integrity and the supportive environment required for germ‐cell development. These findings highlight that orchitis‐associated infertility is not solely driven by germ cell loss, but also involves widespread deterioration of the cellular ecosystem that sustains spermatogenesis.

Beyond structural alterations, we observed extensive remodeling of the testicular immune landscape. The accumulation of cytotoxic CD8^+^ Teff cells, emergence of inflammatory CCL2^+^ TRM cells, and depletion of homeostatic TRM cells suggest a shift from immune surveillance toward a sustained inflammatory state. Such immune imbalance may disrupt blood–testis barrier integrity, promote leukocyte recruitment, and further impair germ‐cell survival and differentiation [43]. Persistent activation of these inflammatory programs may therefore contribute to the long‐term pathological consequences of orchitis.

Our study further revealed concurrent inflammatory and metabolic alterations associated with orchitis pathology. Interstitial cells exhibited coordinated changes in cholesterol metabolism, including increased expression of *SREBF2*, *LDLR*, and *HMGCR*. Given the central role of SREBP2 in cholesterol homeostasis and the reported association between cholesterol metabolic dysregulation and inflammatory signaling [44, 45, 46, 47], our findings suggest that SREBP2‐associated cholesterol metabolic reprogramming represents an important component of orchitis pathology. This highlights orchitis as a complex disorder involving both immune and metabolic disturbances rather than inflammation alone. Therapeutically, our findings provide pharmacological evidence supporting the involvement of the SREBP pathway in inflammation‐associated testicular injury. Fatostatin treatment in the mouse orchitis model improved spermatogenic outcomes and reduced fibrosis and lipid accumulation. Although the relative contributions of individual SREBP isoforms require further investigation, targeting this pathway may represent a complementary strategy to conventional anti‐inflammatory approaches by addressing metabolic abnormalities associated with tissue injury. These findings suggest that modulation of cholesterol metabolic pathways may have broader relevance for inflammation‐associated spermatogenic failure beyond orchitis.

Several limitations should be considered when interpreting these findings. First, the human tissues were obtained during micro‐TESE from patients with orchitis‐induced NOA and therefore represent the chronic post‐orchitis state rather than active infection. Accordingly, these data capture established cellular and molecular alterations associated with prior orchitis‐related injury; however, the cross‐sectional design cannot determine the temporal sequence of pathological changes or distinguish initiating events from secondary alterations during disease progression [48]. Second, the six‐day LPS model represents acute inflammation‐associated injury and does not fully reproduce the viral etiology or complete clinical progression of mumps orchitis. Nevertheless, it recapitulates key pathological features relevant to our human observations, including inflammatory infiltration and spermatogenic impairment, and provides a tractable system for investigating inflammation‐related mechanisms [49]. Third, Fatostatin inhibits SCAP‐dependent activation of both SREBP1 and SREBP2 and may also exert SCAP‐independent effects [50, 51]. Therefore, its therapeutic effects cannot be attributed exclusively to SREBP2. Although the prominent *SREBF2* ‐associated program and relatively low SREBF1 expression support a major contribution of SREBP2, isoform‐specific perturbations will be required to establish their respective causal roles.

In summary, our single‐cell atlas defines orchitis‐associated testicular pathology characterized by loss of undifferentiated spermatogonia, remodeling of the somatic niche, inflammatory immune infiltration, and dysregulated cholesterol metabolism. By integrating human single‐cell profiling with in vivo pharmacological intervention, this study identifies the SREBP pathway as a potential metabolic vulnerability in inflammation‐associated spermatogenic failure and provides a framework for developing targeted strategies to preserve reproductive function.

## 4 Materials and Methods

### 4.1 Clinical Samples and Ethics Statement

Testicular samples from four infertile men with a documented history of post‐pubertal mumps infection complicated by bilateral orchitis at 15–19 years of age were obtained from Peking University Third Hospital for single cell RNA sequencing. Additionally, testicular samples from eight patients with a history of mumps orchitis were included for experimental validation, together with eight patients with obstructive azoospermia serving as controls with preserved spermatogenesis. All 12 patients with a history of mumps orchitis underwent fertility assessment and microdissection testicular sperm extraction (micro‐TESE) between 31 and 36 years of age due to non‐obstructive azoospermia. All cases showed marked bilateral testicular atrophy, with testicular volume of 3–4 mL. Clinical evaluation found no evidence of other genital tract infections, chromosomal abnormalities, known genetic defects, cryptorchidism, varicocele, reproductive tract obstruction, endocrine disorders, or other major identifiable causes of male infertility. These clinical findings support our interpretation that previous bilateral mumps orchitis was the most likely contributor to testicular dysfunction in these patients. These samples were all collected from the discarded tissue during surgical testicular sperm extraction and informed consent was obtained from all the patients before the surgery. The current research was approved by the ethics committee of Peking University Third Hospital (IRB00006761‐M2022692). Concurrently, scRNA‐seq data from four healthy adult males were obtained from a publicly available dataset (GEO: GSE254315 [52], GSE182786 [53]).

### 4.2 Testis Sample Collection

Testicular samples were surgically extracted from patients during microsurgical testicular sperm extraction (micro‐TESE) and were immediately stored on ice after extraction. The tissues were thoroughly washed with PBS and then sliced into small cubes for further processing.

### 4.3 Testis Sample Fixation for Histological Staining

Tissue specimens were first fixed with 4% paraformaldehyde (PFA) for 24 h and subsequently washed three times in 0.01 M PBS, each session lasting 10 min, to ensure complete PFA removal. Dehydration was then carried out using a graded ethanol series followed by xylene immersion. Specifically, tissues were treated sequentially with 50%, 75%, 80%, 95% (twice), and 100% (twice) ethanol, and then transferred to a 1:1 ethanol‐xylene mixture, and finally to pure xylene (twice). The duration for each step ranged from 30 to 50 min and was adaptively modified based on ambient temperature to achieve optimal dehydration. Finally, the tissues were embedded within paraffin and sliced into sections for subsequent histological staining.

### 4.4 H&E Staining

Sections were stained with hematoxylin (Servicebio, G1004) for 8 min until nuclei appeared dark blue‐purple under the microscope, and the reaction was stopped with deionized water. Differentiation was carried out in hematoxylin differentiation solution (Servicebio, G1039) for 20 s, followed by rinsing in deionized water, and bluing was performed in bluing reagent (Servicebio, G1040) for 4 min. The slides were then sequentially immersed in 50%, 70%, and 85% ethanol for 1 min each and in 95% ethanol for 2 min, after which they were stained with eosin (Beyotime, C0105M‐2) for 10 s while carefully monitoring the staining intensity. Following eosin staining, sections were rinsed in deionized water for 5 min and placed in 95% ethanol for 2 min. Finally, the sections were dehydrated in graded ethanol (95% and 100%) and cleared in two changes of xylene for 2 min each, then mounted with neutral resin and air‐dried in a fume hood before storage at room temperature for further observation and analysis.

### 4.5 Masson's Trichrome Staining

In brief, paraffin‐embedded testicular tissue sections (5 µm) were deparaffinized in xylene, rehydrated through a graded ethanol series (100%, 100%, 95%, 80%, and 75%), and rinsed in distilled water. Masson's trichrome staining was then performed using a commercial kit (Masson's Trichrome Stain Kit, Servicebio, G1006) according to the manufacturer's instructions. Finally, the sections were dehydrated, cleared, and mounted with a resinous medium.

### 4.6 Immunofluorescence Staining

Paraffin sections were deparaffinized and subjected to antigen retrieval using citrate buffer (Beyotime, P0083) in a microwave for 10 min, followed by cooling to room temperature. The sections were then washed with PBS on a shaker for 3 × 5 min. After blocking with 50 µL of 5% BSA/PBS at room temperature for 30 min in a humidified chamber, primary antibodies diluted in blocking buffer were applied (50 µL per section) and incubated overnight at 4°C. The primary antibodies used included anti‐CD8 (ab237709, Abcam, 1:100), anti‐CD68 (ab213363, Abcam, 1:100), anti‐FGFR3 (sc‐13121, Santa Cruz Biotechnology; 1:100), anti‐PNA (Invitrogen, L21409; 1:500), anti‐ACTA2 (ab7817, Abcam; 1:1600), anti‐ECM1 (ab253244, Abcam; 1:100), In Situ Cell Death Detection Kit, POD (TUNEL, Roche, 11684817910), anti‐Collagen I (ab34710, Abcam; 1:200), anti‐SOX9 (ab185966, Abcam; 1:300), anti‐SREBP2 (A25685, Abclonal; 1:1600), and anti‐HSD3B2 (15516‐1‐AP, Proteintech; 1:200). The next day, sections were rewarmed for 30 min at room temperature, washed with PBST (3 × 5 min), and incubated with secondary antibodies diluted in PBST (50 µL per section) for 1 h at room temperature in a humidified chamber. After washing with PBST (3 × 5 min), slides were counterstained and mounted with antifade medium containing DAPI (Beyotime, P0131), covered with coverslips, and sealed with nail polish. For co‐staining of anti‐SREBP2, anti‐ACTA2, and anti‐HSD3B2, a four‐color multiplex immunohistochemistry kit (Universal Secondary Antibody, pH 9.0; abs50012, Absin) was used according to the manufacturer's instructions. Following final washes, the slides were cover slipped using Gel‐Mount medium and imaged under a confocal microscope (Zeiss LSM710).

### 4.7 Immunohistochemical Staining

Paraffin sections were deparaffinized and subjected to antigen retrieval using citrate buffer (Beyotime, P0083) in a microwave for 10 min, followed by cooling to room temperature. The sections were then washed with PBS on a shaker for 3 × 5 min. Immunohistochemical staining was performed using the Mouse and Rabbit Specific HRP/DAB Detection IHC Kit (Abcam, ab64264) according to the manufacturer's instructions. Primary antibodies, including anti‐SREBP2 (A25685, Abclonal; 1:1000), anti‐LDLR (ab52818, Abcam; 1:500), and anti‐ABCA1 (NB400‐105, Novus Biologicals; 1:200), were incubated with the sections overnight at 4°C. After rewarming the following day, the staining procedure was continued in accordance with the kit protocol. Finally, the sections were dehydrated, cleared, and mounted with a resinous medium.

### 4.8 Oil Red O Staining

Following fixation, testis tissues were equilibrated through a sucrose–PBS gradient (10%, 15%, 20%) and embedded in OCT medium (Tissue‐Tek, cat. 4583) at −80°C prior to cryosectioning. Frozen sections were placed at −20°C for 30 min, then equilibrated at room temperature for 30 min, and subsequently washed with PBS on a shaker three times for 5 min each. Oil Red O staining was performed according to the manufacturer's instructions using the Modified Oil Red O Staining Kit (Beyotime, C0158M).

### 4.9 Animal

Male ICR mice aged 8 weeks were obtained from the Institutional Animal Care and Use Committee of Institute of Zoology, Chinese Academy of Sciences and housed under specific pathogen‐free (SPF) conditions. The animals were housed in individually ventilated cages (four per cage) under a 12‐h light/dark cycle at 23.0 ± 3.0 °C, with ad libitum access to standard rodent chow and water.

Mice were randomly assigned to three experimental groups: control, orchitis, and rescue. All treatments were administered once daily. For the first three days, control group received intraperitoneal injections of saline, while mice in the mouse orchitis and rescue groups were administered 2.5 mg/kg lipopolysaccharide (LPS; Sigma, L2880) intraperitoneally to induce inflammation. From days 4 to 6, the control group continued to receive saline, the mouse orchitis group was treated with corn oil (MCE, HY‐Y1888), and the rescue group received 20 mg/kg Fatostatin (MCE, HY‐14452). At the end of the treatment regimen, all mice were euthanized, and testes and epididymides were collected, weighed, and processed for histological examination and molecular analyses.

### 4.10 Sperm Count and Motility Assessment

The caudal epididymides of mice were incised into three segments and placed in human tubal fluid (HTF) medium at 37 °C for 5 min to allow sperm release. 10 µL of the resulting sperm suspension was analyzed using a computer‐assisted semen analysis (CASA) system (SSA‐II Gold Edition, SuiJia Software, China) to evaluate sperm concentration and motility, following World Health Organization guidelines. The assessed parameters included sperm concentration (10^6^/mL), progressive motility (%, PR), and total motility (%, PRNP). For each sample, a minimum of 200 spermatozoa were examined to ensure statistical reliability.

### 4.11 Western Blotting

Testicular protein extracts were prepared by mechanical homogenization in ice‐cold RIPA buffer (Beyotime) supplemented with 1 mM PMSF. Following clarification by centrifugation at 12 000 × g for 10 min at 4°C, the supernatants were retained and total protein content was quantified using a BCA assay kit (Pierce/Thermo Scientific). For immunoblotting, 10 µg of protein from each sample was mixed with loading buffer, denatured, and subjected to electrophoretic separation on 10% SDS–PAGE gels. Proteins were then electrotransferred onto PVDF membranes (Bio‐Rad, Hercules, CA, USA). After transfer, membranes were incubated for 1 h at room temperature in TBST containing 5% non‐fat dry milk to minimize nonspecific binding. Immunodetection was performed by overnight incubation at 4°C with antibodies against SREBP2 (28212‐1‐AP, Proteintech; 1:1,000) and β‐actin (YM3028, ImmunoWay Biotechnology; 1:3,000). After extensive washing, species‐matched HRP‐conjugated secondary antibodies were applied at a dilution of 1:5,000, including goat anti‐rabbit IgG (BF03008) and goat anti‐mouse IgG (BF03001; Biodragon Immunotechnologies). Chemiluminescent signals were developed using an enhanced chemiluminescence reagent (New Cell & Molecular Biotech) and captured with a Tanon 5200 imaging system (Tanon, Shanghai, China). Densitometric analysis of the immunoreactive bands was carried out using ImageJ software (NIH), with β‐actin serving as the loading control.

### 4.12 Testosterone Detection by ELISA

Serum and intratesticular testosterone concentrations were determined using a commercial mouse testosterone (T) ELISA kit (FM43305, FeiMoBio, Beijing, China). Testicular tissues were homogenized in physiological saline and centrifuged at 3,000 rpm for 10 min, and the supernatants were collected for analysis. Samples and testosterone standards (0–800 pg/mL) were incubated with HRP‐conjugated detection antibody for 60 min at 37°C. After five washes, TMB substrates were added and incubated for 15 min at 37°C in the dark. The reaction was terminated with stop solution, and absorbance was measured at 450 nm. Testosterone concentrations were calculated from the corresponding standard curve and corrected for the sample dilution factor.

### 4.13 Total Cholesterol Measurement

Total cholesterol levels in testicular tissues were quantified using a Tissue Total Cholesterol Content Assay Kit (E1015, Applygen Technologies, Beijing, China) according to the manufacturer's instructions. Briefly, testicular tissues were homogenized in the supplied lysis buffer and centrifuged at 2 000 rpm for 5 min. The resulting supernatants were incubated with the cholesterol detection working solution at 37°C for 20 min, and absorbance was measured at 550 nm. TC concentrations were calculated from a cholesterol standard curve and normalized to the corresponding protein concentration.

### 4.14 Tissue Preparation for Single Cell RNA Sequencing

For each single‐cell RNA‐seq experiment, tissue samples were rapidly thawed, rinsed twice with PBS, and mechanically dissociated using razor blades. Tissue fragments were then subjected to a two‐step enzymatic digestion process: first with 1 mg/mL collagenase type IV (C8160, Solarbio) and 1 mg/mL DNase I (D8071, Solarbio), followed by treatment with trypsin‐EDTA (T1300, Solarbio) and 1 mg/mL DNase I at 37 °C for 5 min. The resulting cell suspension was filtered through a 70 µm mesh strainer (Miltenyi, 130‐098‐462) and washed with D‐PBS (GIBCO, 14040133). Finally, cells were resuspended in D‐PBS containing 0.4% BSA (Sigma, B2064) at a concentration of approximately 1,000 cells/µL for subsequent single‐cell RNA sequencing.

### 4.15 Single Cell RNA‐Seq Library Preparation and Sequencing

The experiment was performed according to the instructions of 10X Genomics using the Chromium Next GEM Single Cell 3′ Reagent Kits v3.1. Cell suspensions were diluted to approximately 5,000 cells per lane and combined with master mix before being loaded onto the Chromium Next GEM Chip G for Gel Bead‐in‐Emulsion (GEM) generation and barcoding. After reverse transcription within GEMs and post‐GEM‐RT cleanup, cDNA was amplified through 12 cycles of PCR. Final libraries were constructed and sequenced on an Illumina NovaSeq 6000 system (Illumina) with the following read configuration: 28 cycles for Read 1, 10 cycles each for i5 and i7 indices, and 90 cycles for Read 2.

### 4.16 Processing of Single Cell RNA‐Seq Data

CellRanger v7.0.0 was used to demultiplex raw data using the mkfastq function. The output FASTQ files were then run with function count using default settings, including alignment to the GRCh38 human reference genome. Subsequently, the resulting matrices were imported into R v.4.2.2. Using the Read10X function in Seurat (v4.3.2), UMI count matrices from 8 individual samples were imported into R. Sample identifiers were incorporated into the matrix, and matrices were merged into a unified Seurat object. Features with fewer than 500 or more than 8,000 features, UMIs with fewer than 600 or more than 30,000 counts and cells with greater than 18% mitochondrial mapping were filtered out. Data were normalized as recommended in the Seurat workflow. Samples from controls (n = 4) and orchitis (n = 4) groups were integrated using the Harmony package (v1.2.3), yielding a batch‐corrected dataset. Dimensionality reduction was performed using PCA, and the top 2000 highly variable genes along with the first 26 principal components were used for UMAP visualization and graph‐based clustering. Cell type annotation was conducted following established marker‐based strategies. For subpopulation analysis, subsets of cells were extracted by annotated cell identity to create new Seurat objects.

### 4.17 Differentially Expressed Genes and Functional Enrichment Analysis

Differentially expressed genes (DEGs) between the control and orchitis groups were identified through the *FindMarkers* function in Seurat, with the thresholds set as |avg_log2FC| > 0.25 and adjusted p value < 0.05. Functional enrichment analysis, including Gene Ontology (GO) and KEGG pathway analyses, was conducted using the clusterProfiler R package.

To quantify the activity of specific biological processes or functional states at the single‐cell level, gene signature scores were calculated using the AddModuleScore function in Seurat based on predefined gene sets. Gene sets obtained from the Molecular Signatures Database (MSigDB, version 2025.1) included WP_CHOLESTEROL_METABOLISM, REACTOME_CHOLESTEROL_BIOSYNTHESIS, GOBP_CELL_CYCLE, and HALLMARK_INFLAMMATORY_RESPONSE. Visualization of differential expression and functional enrichment results was supported by multiple R packages, including ClusterGVis (v0.1.1), ggstatsplot (v0.12.1), ggkegg (v1.0.5), and ggplot2 (v3.4.4).

### 4.18 Principal Component Analysis

Principal Component Analysis (PCA) was conducted to visualize sample relationships based on single‐cell RNA sequencing data. The top 200 marker genes were identified using the *FindAllMarkers* function in Seurat, and their average expression values were computed with the *AverageExpression* function. The resulting 2D/3D PCA projections were visualized using the plotly package, with individual samples distinctly colored to facilitate comparative analysis.

### 4.19 Cell‐Cell Communication Analysis

CellChat (https://github.com/sqjin/CellChat, v1.6.1) was applied for cell‐cell communication analysis. CellChat object was created from the gene expression matrix and cell metadata using the *createCellChat* function. The “Secreted Signaling” ligand–receptor database was employed for subsequent analysis. Gene expression data were projected onto a protein–protein interaction network using methods recommended in the package tutorial. Communication probabilities were computed with the *computeCommunProb* function, and pathway‐level interactions were inferred via the *computeCommunProbPathway* function. The signaling patterns of intercellular interactions were further identified using *identifyCommunicationPatterns* function.

### 4.20 SCENIC Analysis

Transcription factor regulatory networks were inferred using SCENIC (Single‐Cell rEgulatory Network Inference and Clustering). Briefly, the single‐cell gene expression matrix was used to infer co‐expression modules between transcription factors and their candidate target genes using GENIE3. The resulting TF–target modules were subsequently subjected to cis‐regulatory motif enrichment analysis using RcisTarget, and modules containing significantly enriched corresponding TF‐binding motifs were retained as regulons. The activity of each regulon in individual cells was then quantified using AUCell based on the enrichment of regulon target genes within the ranked gene‐expression profile of each cell. Regulon activity scores were compared across cell populations to identify cell‐type‐ and disease‐associated transcriptional regulators.

### 4.21 STARTRAC‐Dist Analysis

The preferential distribution of cell populations between control and orchitis samples was evaluated using the STARTRAC‐distribution (STARTRAC‐dist) index. A contingency table was constructed according to cell type and disease group, and the expected number of cells for each cell type–group combination was estimated under the assumption of random distribution. The STARTRAC‐dist index was calculated as the ratio of the observed to expected cell number (Ro/e) for each cell population in each group. An Ro/e value > 1 indicated relative enrichment of the corresponding cell population in a given group, whereas an Ro/e value <1 indicated relative depletion. The resulting STARTRAC‐dist indices were used to characterize disease‐associated alterations in testicular cell composition.

### 4.22 Statistical Analysis

Data were presented as mean ± SD for normally distributed data or as median (interquartile range) for non‐normally distributed data, as detailed in the figure legends. All statistical analyses were performed using R Statistical Software (v4.4.2). Data normality was assessed using the Shapiro‐Wilk test. For comparisons between two groups, a two‐tailed unpaired Student's t‐test was applied to normally distributed data, whereas the Wilcoxon rank‐sum test was used for non‐normally distributed data. For comparisons involving more than two groups, statistical significance was determined using one‐way analysis of variance (ANOVA) followed by Tukey's post hoc test, or using the Kruskal‐Wallis test when data did not meet normality assumptions. When the Kruskal‐Wallis test was significant, pairwise comparisons were performed using the Wilcoxon rank‐sum test with Benjamini‐Hochberg correction for multiple comparisons. Significance levels are indicated as follows: *p < 0.05; **p < 0.01; ***p < 0.001; ****p < 0.0001.

## Author Contributions

Q.L., J.G., Z.Zhang, and H.J. conceived the study. Y.T. and Q.J. performed computational data analysis. X.W., W.Z., L.C., X.L. and Z.Zhuang assisted with data interpretation. Q.L., Y.T., Z.Zhang, K.H., J.Q. and H.J. performed scRNA‐seq library construction and sequencing. Y.T., Q.J., Y.L., J.C., Y.F. and S.G. performed histological experiments and confocal photography. Y.T., Q.J., J.G., and Q.L. wrote the manuscript with input from all authors.

## Conflicts of Interest

The authors declare no conflicts of interest.

## Supporting information




**Supporting File 1**: advs78572‐sup‐0001‐SuppMat.pdf.

**Supporting File 2**: advs78572‐sup‐0002‐TableS1.xlsx.

## Data Availability

The raw sequencing data generated in this study have been deposited in the Genome Sequence Archive at National Genomics Data Center, China National Center for Bioinformation/Beijing Institute of Genomics, Chinese Academy of Sciences (https://ngdc.cncb.ac.cn/gsa‐human, accession no. HRA006586 and HRA014419) for scRNA‐seq data. The healthy scRNA‐seq data used in this study are publicly available in the GEO database under accession codes GSE254315^52^ and GSE182786^53^.
