# Supplementary material for: Single‐Cell Insights Into Pathogenic Profile of Orchitis and SREBP2‐Associated Cholesterol Dysregulation in Spermatogenic Failure
Source: Adv Sci (Weinh). 2026 Oct 31:e78572. Online ahead of print. doi: 10.1002/advs.78572 (PMC13723723; doi:10.1002/advs.78572)
Supplement: Supplementary file 1 [file ADVS-9999-e78572-s001.pdf]

Supporting Information

Single-cell insights into pathogenic profile of orchitis and SREBP2-associated cholesterol dysregulation in spermatogenic failure

Yanlin Tang, Qiaoling Jiang, Yuwen Liu, Wenxiu Zhang, Lina Cui, Xiaojian Lu, Ziyan Zhuang, Jianxing Cheng, Songzhan Gao, Yangyi Fang, Kai Hong, Jie Qiao, Xiaoyan Wang, Hui Jiang, Zhe Zhang*, Jingtao Guo*, Qiang Liu**

**Correspondence:*
Qiang Liu (lqiang1210@bjmu.edu.cn),
Jingtao Guo (jingtao.guo@ioz.ac.cn),
Zhe Zhang (zhezhang@bjmu.edu.cn),
Hui Jiang (jianghui@bjmu.edu.cn)

Fig.S1 Single-cell transcriptome profile of testicular cells from healthy men and orchitis.

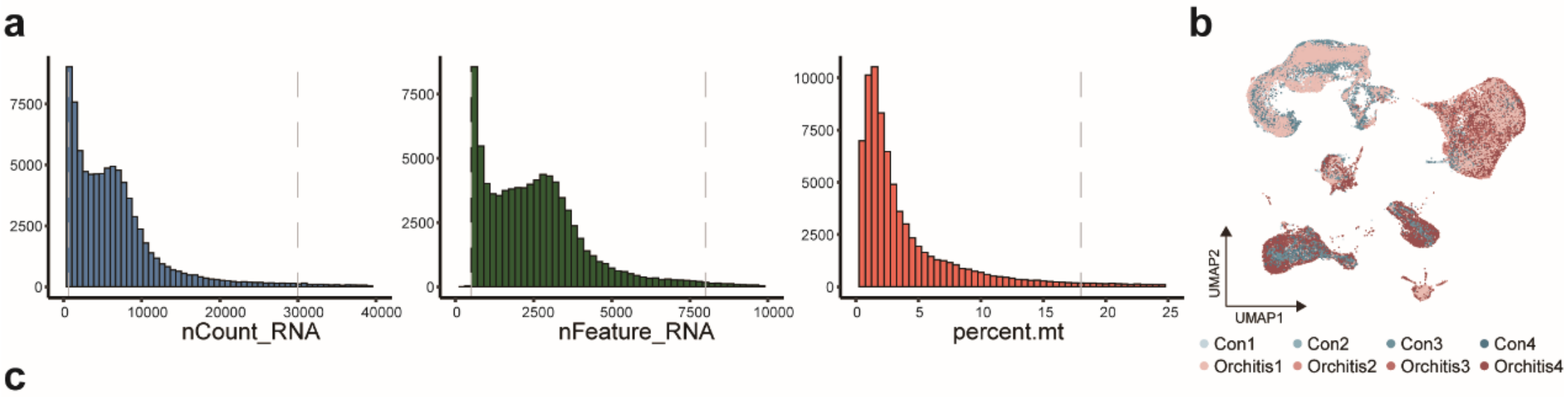

c

Doner_ID	Diagnosis	Karyotype	Comorbidities	Age	PRL (ng/ml)	FSH (IU/L)	LH (IU/L)	T (nmol/L)	Testicular size (ml)
Orchitis1	Mumps orchitis induced NOA	46, XY	None	34	15	31	16	2.87	3ml
Orchitis2	Mumps orchitis induced NOA	46, XY	None	34	8.6	50.4	12.1	6.41	4ml
Orchitis3	Mumps orchitis induced NOA	46, XY	None	31	19	26.7	11.1	8.25	4ml
Orchitis4	Mumps orchitis induced NOA	46, XY	None	36	-	21.27	7.13	2.91	4ml

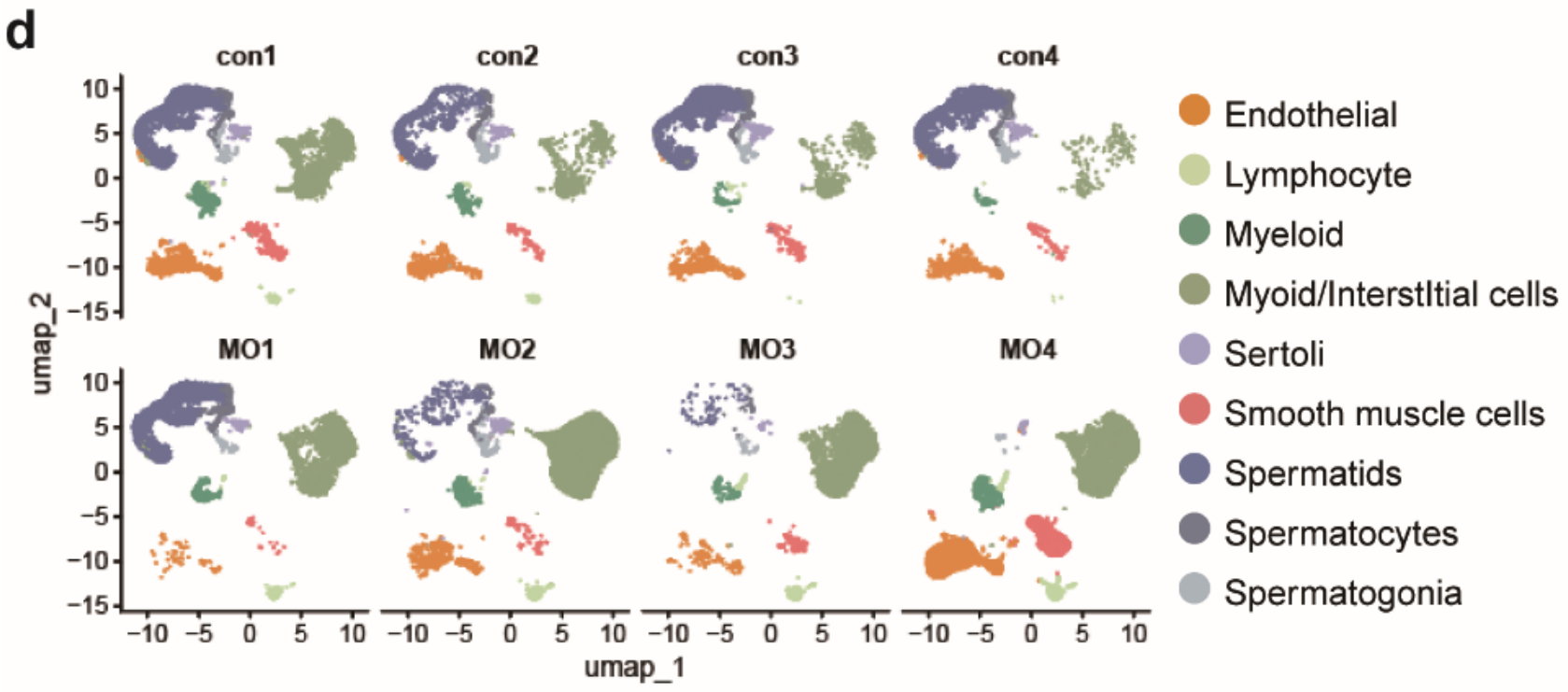

Figure S1 Single-cell transcriptomic atlas of testicular cells from healthy men and orchitis.

a. Histogram displaying the number of count RNA and feature RNA and the mitochondrial RNA percentage of the four control and four orchitis data.

b. UMAP plots of the testis sample from controls (Con1-4) and orchitis (Orchitis 1-4) with colors representing different samples.

c. Table showing the clinical information (age, hormone levels and testicular size) of the four orchitis patients.

d. UMAP plots of four control and four orchitis samples, with each sample displayed in a separate UMAP plot and cells colored by cell type.

Fig.S2 Molecular signatures of immune cells in orchitis.

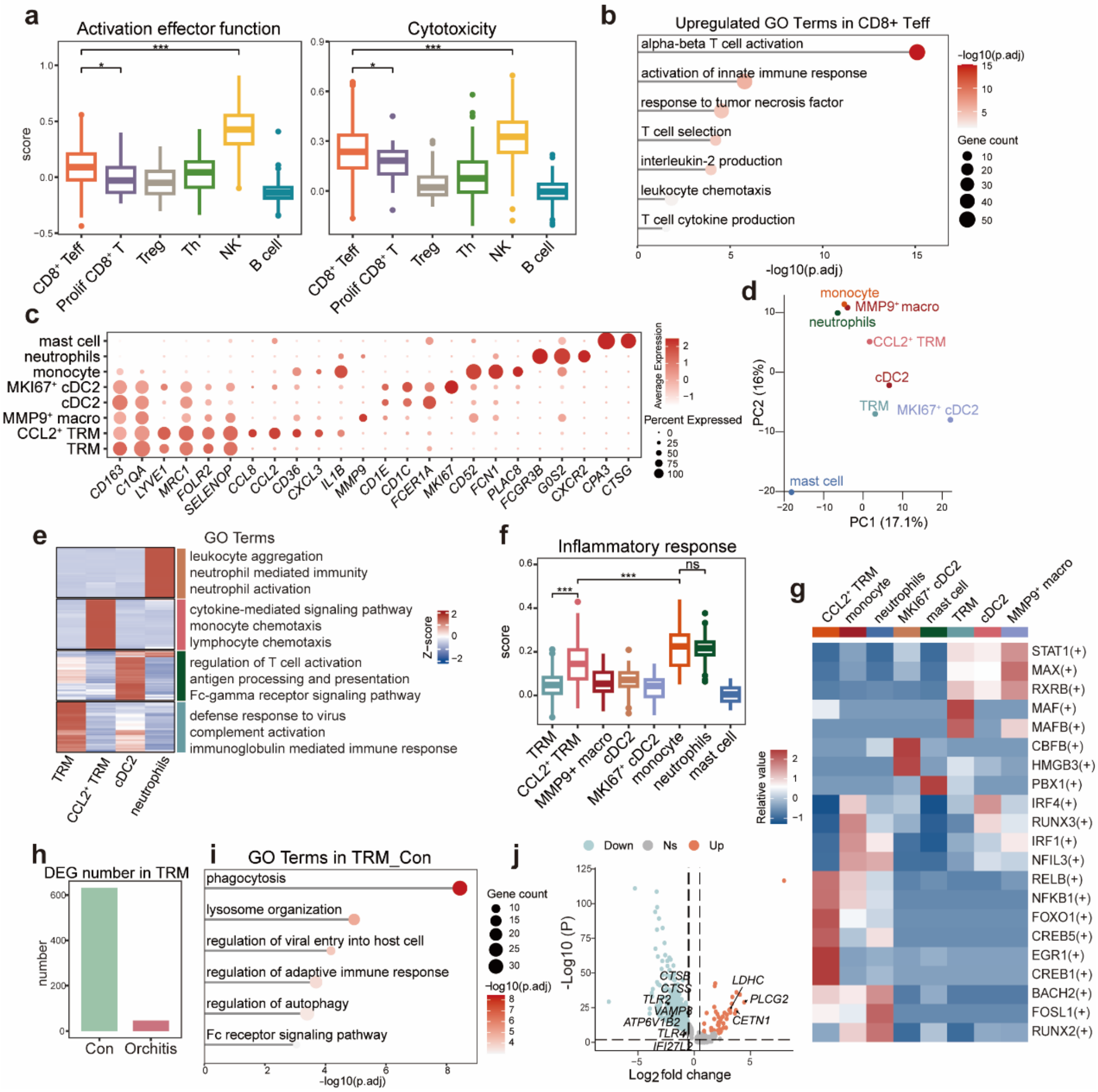

Figure S2 Molecular signatures of immune cells in orchitis.

a. Box plots showing the signature scores of activation/effector function (left) and cytotoxicity (right) across lymphocyte subtypes. Color indicates different lymphocyte subtypes. Boxes indicate the interquartile range (25th–75th percentile), center lines indicate the median, and dots represent individual cells. The P value was calculated using the Wilcoxon test. *$p < 0.05$, *** $p < 0.001$.

b. The upregulated GO terms enriched in the CD8$^+$ Teff cells of the orchitis group compared to that in the control group.

c. Dot plot presenting the expression level of selected markers in each myeloid cell. Dot size represents the expression ratio of each gene and the gradient of orange color represents the average expression level of the genes.

d. PCA plot displaying the distribution of myeloid subtypes.

e. Heatmap showing the gene expression patterns of TRM, $CCL2^{+}$ TRM, cDC2 and neutrophils (left) with the corresponding enriched GO terms (right). The color gradients represent the gene expression z-score as the legend to the right.

f. Box plot showing the signature score of inflammatory response in different myeloid cells. Color indicates the myeloid subtypes, box indicates range from 25th to 75th percentile; center lines indicate the medium, dots represent individual cells. The p value was calculated by Wilcoxon test. ***$p < 0.001$. ns, not significant.

g. Heatmap displaying the expression pattern of the key transcription factors in different myeloid cells.

h. The number of DEGs in the TRM cells of the control and orchitis groups.

i. Bar plot displaying the GO terms enriched in the TRM cells of the control group.

j. Volcano plot presenting the differentially expressed genes of the TRM cells between the control and orchitis group. Each dot represents one gene and different colors represent different expression patterns as shown in the legend at the top.

Fig.S3 Intercellular signaling may drive immune infiltration in orchitis.

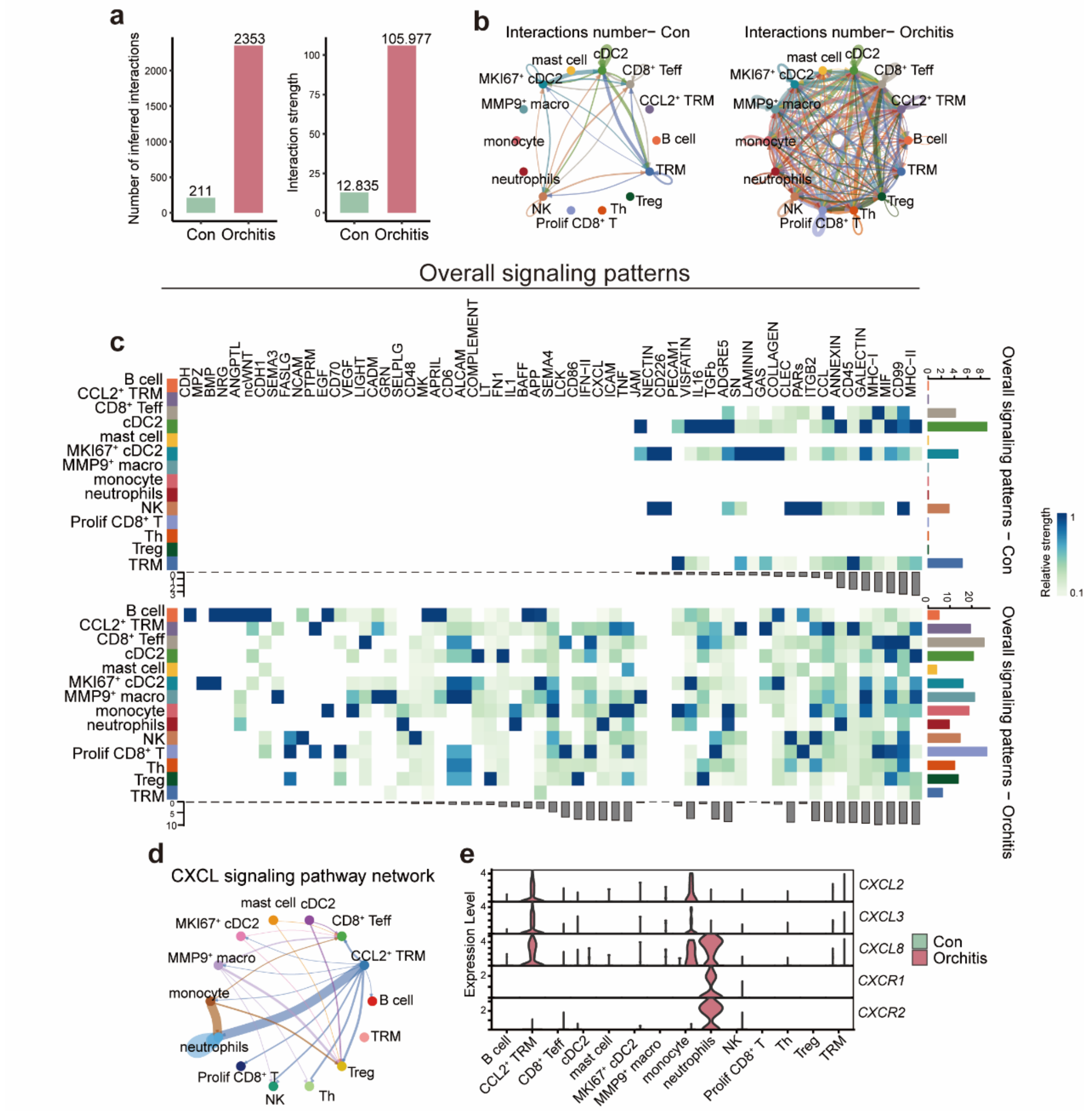

Figure S3 Intercellular signaling may drive immune infiltration in orchitis.

a. Bar plot showing the number (left) and the strength (right) of the inferred cell-cell interaction in the controls and orchitis groups.

b. Circle plots demonstrating the numbers of cell-cell interaction in the control (left) and orchitis (right) groups.

c. Heatmap illustrating the overall cell-cell signaling patterns among different types of immune cells in the control (top) and orchitis (bottom) groups. The color gradients represent the relative strength of signaling with dark blue meaning high strength.

d. Circle plot displaying the CXCL signaling pathway network among different immune cells in orchitis.

e. Violin plots showing the differential expression of CXCL signaling–related ligands and receptors in different immune cells of the control and orchitis groups.

Fig.S4 Disrupted spermatogenic niche and interstitial cell dysfunction in orchitis.

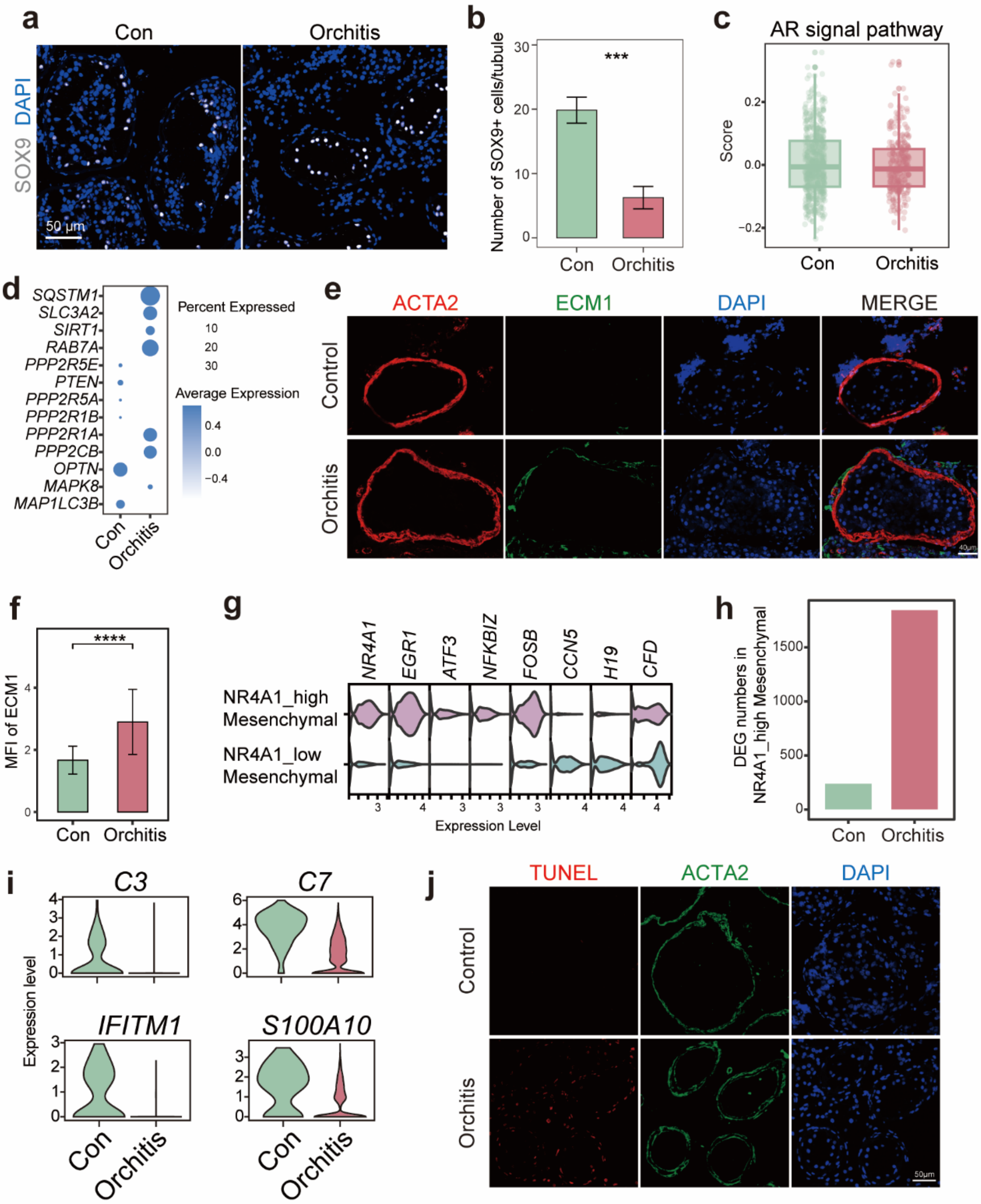

Figure S4 Disrupted spermatogenic niche and interstitial cell dysfunction in orchitis.

a. IF staining of SOX9 (light grey) in the tissue section of control and orchitis groups. Scale bar, 50μm.

b. Quantification of the number of $SOX9^+$ cells per spermatogenic tubule in the control and orchitis groups. Bars represent the mean with SD of 30 independent tubules in the control (n=3) and orchitis groups (n=3). The p value was calculated by Student's t test. ***p<0.001.

c. Bar plot showing the signature score of AR signal pathway in the control and orchitis groups. Box indicates range from 25th to 75th percentile; center lines indicate medium, dot represent individual cells.

d. Dot plot presenting the expression level of phagocytosis-related genes in Sertoli cells from control and orchitis groups. Dot size represents the expression ratio of each gene and the gradient of blue color represents the average expression level of the genes.

e. IF staining of ECM1 (green) and ACTA2 (red) in the tissue section of the control and orchitis group. Scale bar, 40μm.

f. Quantification of the mean fluorescence intensity of ECM1 per spermatogenic tubule in the control and orchitis groups. Bars represent the mean with SD of 30 independent tubules in the control (n=3) and orchitis groups (n=3). The p value was calculated by Student's t test. ***p<0.001.

g. Violin plot showing the expression of select marker genes of NR4A1_high and NR4A1_ low mesenchymal.

h. The number of DEG in the NR4A1_high mesenchymal cells of the control and orchitis groups.

i. Violin plot representing the differential expression of inflammation-related genes in the NR4A1_high mesenchymal cells from control and orchitis groups.

j. IF staining of TUNEL (Red), ACTA2 (Green) and DAPI (Blue) in orchitis and control group. Scale bar, 50μm.

Fig.S5 SREBP2-associated cholesterol accumulation in interstitial cells of human and mouse orchitis.

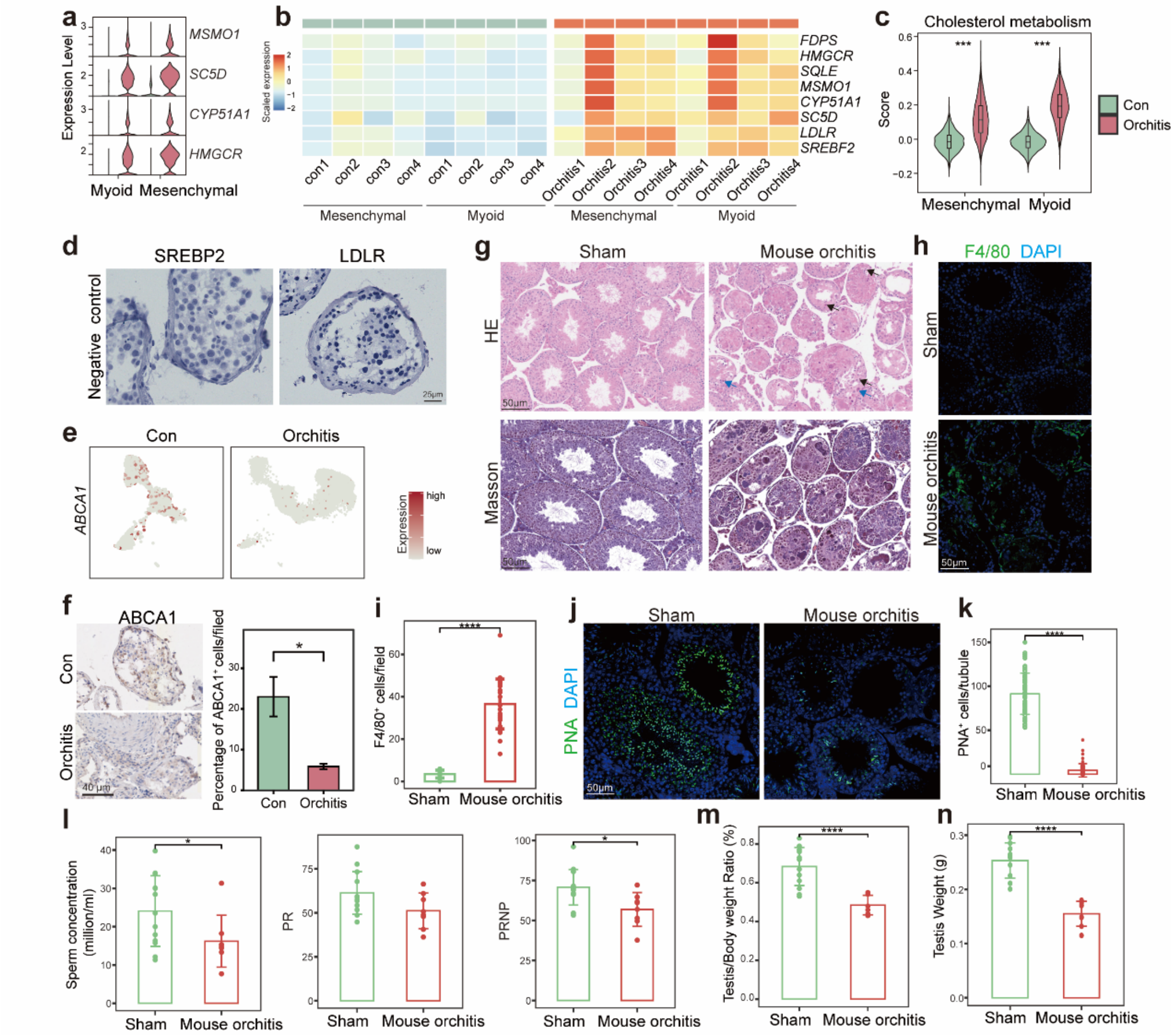

Figure S5 SREBP2-associated cholesterol accumulation in interstitial cells of human and mouse orchitis.

a. Violin plot representing the expression levels of cholesterol synthesis related genes in the myoid cells and mesenchymal cells of the control and orchitis groups.

b. Heatmap illustrating the expression patterns of cholesterol synthesis related genes in the myoid cells and mesenchymal cells in each sample.

c. The calculated scores of cholesterol metabolism in the mesenchymal cells and myoid cells of the control and orchitis groups. The p value was calculated by Student's t test. ***$p<0.001$.

d. Negative controls for the IHC staining of SREBP2 and LDLR. Scale bar, 25µm.

e. The expression of ABCA1 in the control and orchitis groups shown by UMAP plot.

f. (left) IHC staining of ABCA1 in the tissue sections of the control (n=3) and orchitis groups (n=3). (right) Quantification of the number of $ABCA1^{+}$ cells per field in the control and orchitis groups. The *p* value was calculated by Student's t test. *$p < 0.05$.

Scale bar, 40µm.

g. H&E staining (top) and Masson staining (bottom) of the testis sections from the sham (n=13) and mouse orchitis groups (n=8). Black arrows point to lymphocytes; blue arrows point to myeloid cells.

h. IF staining of F4/80 (green) in the tissue section of sham and mouse orchitis groups. Scale bar, 50μm.

i. Quantification of the number of F4/80^{+} macrophage cells per field in the sham and mouse orchitis groups. Bars represent the mean with SD of 30 independent fields in the sham (n=3) and orchitis groups (n=3).

j. IF staining of PNA in the tissue sections of the sham and mouse orchitis groups. Scale bar, 50μm.

k. Quantification of PNA^{+} cells per spermatogenic tubule in the sham (n=13) and mouse orchitis groups (n=8). The p value was calculated by Student's t test. ****$p<0.0001$.

l. Histogram demonstrating the differences of sperm quality indicators (sperm concentration, PR, PRNP) between the sham (n=13) and mouse orchitis groups (n=8). PR, progressive motility. PRNP, progressive and non-progressive motility. The p value was calculated by Student's t test. *$p<0.05$.

m-n. Histogram displaying the differences of testis/body weight ratio (j) and the testis weight (k) between the sham (n=13) and mouse orchitis groups (n=8). The p value was calculated by Student's t test. ****$p<0.0001$.

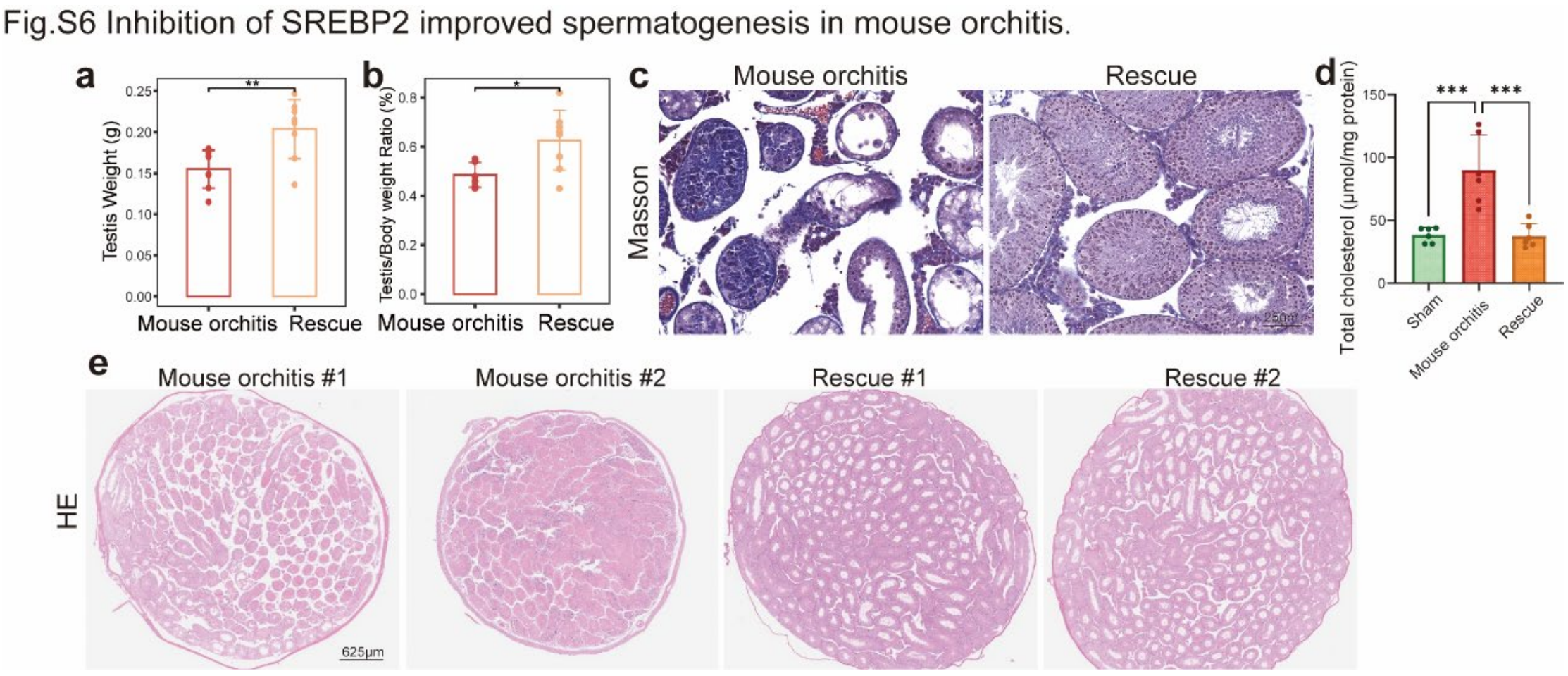

Figure S6 Inhibition of SREBP2 improved spermatogenesis in mouse orchitis.

a-b. Histogram displaying the differences of testis weight (a) and the testis/body weight ratio (b) between the mouse orchitis (n=8) and rescue groups (n=8). The p value was calculated by Student’s t test. **$p<0.01$. *$p<0.05$.

c. Masson staining of testis sections from the mouse orchitis (n=8) and rescue groups (n=8). Scale bar, 25μm.

d. Quantification of total cholesterol in testicular tissue from the sham, mouse orchitis and rescue groups, n = 6 per group. All data are presented as mean ± SD and data are analyzed by One-way ANOVA. **, $p < 0.01$; ****, $p < 0.0001$.

e. H&E staining of the testis sections from two mouse orchitis samples and two rescue samples. Scale bar, 625μm.
